# Intake of Protein Plus Carbohydrate during the First Two Hours after Exhaustive Cycling Improves Performance the following Day

**DOI:** 10.1371/journal.pone.0153229

**Published:** 2016-04-14

**Authors:** Per I. Rustad, Manuela Sailer, Kristoffer T. Cumming, Per B. Jeppesen, Kristoffer J. Kolnes, Ove Sollie, Jesper Franch, John L. Ivy, Hannelore Daniel, Jørgen Jensen

**Affiliations:** 1 Department of Physical Performance, Norwegian School of Sport Sciences, P.O. Box 4014 Ullevål Stadion, N-0806 Oslo, Norway; 2 ZIEL Institute for Food and Health, Technische Universiät München, Munich, Germany; 3 Department of Endocrinology and Internal Medicine, Aarhus University Hospital, Aarhus University, Aarhus, Denmark; 4 Department of Health Science and Technology, Aalborg University, Ålborg, Denmark; 5 Exercise Physiology and Metabolism Laboratory, Department of Kinesiology and Health Education, University of Texas at Austin, Austin, United States of America; 6 Department of Nutrition, Exercise and Sports, University of Copenhagen, Copenhagen, Denmark; Victoria University, AUSTRALIA

## Abstract

Intake of protein immediately after exercise stimulates protein synthesis but improved recovery of performance is not consistently observed. The primary aim of the present study was to compare performance 18 h after exhaustive cycling in a randomized diet-controlled study (175 kJ·kg^-1^ during 18 h) when subjects were supplemented with protein plus carbohydrate or carbohydrate only in a 2-h window starting immediately after exhaustive cycling. The second aim was to investigate the effect of no nutrition during the first 2 h and low total energy intake (113 kJ·kg^-1^ during 18 h) on performance when protein intake was similar. Eight endurance-trained subjects cycled at 237±6 Watt (~72% VO_2max_) until exhaustion (TTE) on three occasions, and supplemented with 1.2 g carbohydrate·kg^-1^·h^-1^ (CHO), 0.8 g carbohydrate + 0.4 g protein·kg^-1^·h^-1^ (CHO+PRO) or placebo without energy (PLA). Intake of CHO+PROT increased plasma glucose, insulin, and branch chained amino acids, whereas CHO only increased glucose and insulin. Eighteen hours later, subjects performed another TTE at 237±6 Watt. TTE was increased after intake of CHO+PROT compared to CHO (63.5±4.4 vs 49.8±5.4 min; p<0.05). PLA reduced TTE to 42.8±5.1 min (p<0.05 vs CHO). Nitrogen balance was positive in CHO+PROT, and negative in CHO and PLA. In conclusion, performance was higher 18 h after exhaustive cycling with intake of CHO+PROT compared to an isocaloric amount of carbohydrate during the first 2 h post exercise. Intake of a similar amount of protein but less carbohydrate during the 18 h recovery period reduced performance.

## Introduction

Insufficient recovery of performance capacity after exhaustive endurance exercise may determine the final outcome in cycling competitions like the Tour de France with consecutive racing days. It is well-documented that muscle glycogen is the major energy substrate during high intensity endurance exercise and it is also known that fatigue develops when muscle glycogen is depleted [1–4]. A high glycogen content in muscles is thus associated with improved performance [5] and a repletion of glycogen stores is part of the recovery process after prolonged exhaustive exercise [6]. Optimal glycogen repletion occurs with an intake of 1.2 g carbohydrate · kg^-1^ · h^-1^ during the first hours and a total of 8–9 g carbohydrate · kg^-1^ during 24 h, which is now accordingly recommended [7,8].

Protein is normally not considered as an important energy substrate during exercise, although oxidation of leucine is known to increase during exercise [9,10]. Furthermore, protein degradation is increased in glycogen-depleted muscles during exercise [11–13], and protein breakdown continues in skeletal muscle after exercise if energy intake is not sufficient or if only carbohydrates are provided [14]. Recovery of performance includes the removal and degradation of proteins damaged during exercise along with the proper resynthesis of proteins.

Dietary intake of branched chain amino acids (BCAA) after exercise reduces loss of amino acids from muscles and therefore spares muscle protein from degradation [15]. Intake of protein immediately after exercise has also consistently shown to increase protein synthesis [16,17], including synthesis of mitochondrial proteins [18], while simultaneously protein degradation is decreased [17]. Importantly, it has been shown that protein intake in the early phase after intensive exercise stimulates protein synthesis more effectively than later [19]. When intake of proteins is combined with carbohydrate after exercise, activation of hypertrophic signalling molecules in skeletal muscles is achieved [20]. Protein intake is necessary for optimal recovery after exhaustive exercise [21,22] requiring both glycogen and protein synthesis [22]. Moreover, there is evidence that the combination of carbohydrates with protein also increases the rate of glycogen synthesis [21,23–25].

Despite the proven effects of protein intake on protein synthesis rate, supplementation of proteins in addition to carbohydrates is not consistently associated with improved exercise performance. Several studies have reported that intake of protein after exercise improves subsequent performance [20,21,26–29] whereas other studies failed to observe such effects [30–33].

These discrepancies may result from differences in the amount of protein supplied, its composition or that the exercise models used may not be adequate to detect the effects of protein intake on recovery of performance. Quite different protocols regarding intensity and duration prior to the diet intervention, and to assess performance after the intervention period have been used. An experimental design with similar exercise intensity prior to and after the diet intervention would represent a more appropriate approach for getting more coherent outcomes assuming that the physiological mechanisms causing fatigue during the initial bout of exhaustive exercise would limit performance capacity during the post exercise test.

In the present study, subjects were exercised to exhaustion at a load corresponding to ~72% VO_2max_ (Watt_72%_), which depletes glycogen stores and such exercise has been used to demonstrate a positive effect of protein intake on recovery of performance [21,26]. The primary aim of the present study was to test the hypothesis that protein/carbohydrate feeding during the first 2 hours after exhaustive exercise is superior to intake of an isocaloric amount of carbohydrate alone on recovery of performance 18 h later. Therefore, we performed a randomized, double-blinded, diet-controlled study in which subjects were given carbohydrate (1.2 g · kg^-1^ · h^-1^) or protein/carbohydrate (0.4 g/0.8 · kg^-1^ · h^-1^) during the first 2 h after exhaustive exercise with diet during the remainder of the recovery period standardized. Nitrogen balance, plasma glucose, amino acids and hormones were measured during recovery and an exercise performance test conducted the following day to determine whether changes in performance after protein intake immediately after exhaustive exercise are associated with distinct changes in energy and amino acid homeostasis.

To test the sensitivity of our protocol selected for investigating exercise performance recovery, we also investigated the effect of insufficient energy intake during recovery, which incompletely restores glycogen content [34]. Therefore, the second aim was to investigate if lack of energy intake during the first 2 h after exhaustive endurance exercise, and a ~50% reduction of total carbohydrate intake, reduces exercise performance after the 18 h recovery period.

## Materials and Methods

### Subjects

Eight male endurance-trained subjects using cycling as part of their weekly training participated in the study (age: 24±0.4 years; height: 182±3 cm; weight 75±3 kg; cycle training 2.9 ±0.6 h weekly). Maximal oxygen uptake was 5.2 ± 0.1 L · min^-1^ (69.6 ±1.3 ml · kg^-1^ · min^-1^). The study was approved by the Regional Committees for Medical and Health Research Ethics (2010/1537/REK sør-øst D), Norway. Participants obtained verbal and written information about the study and signed informed consent to participate. Informed consent was approved by ethics committee and signed forms are stored.

### Experimental design

**Fig 1:**
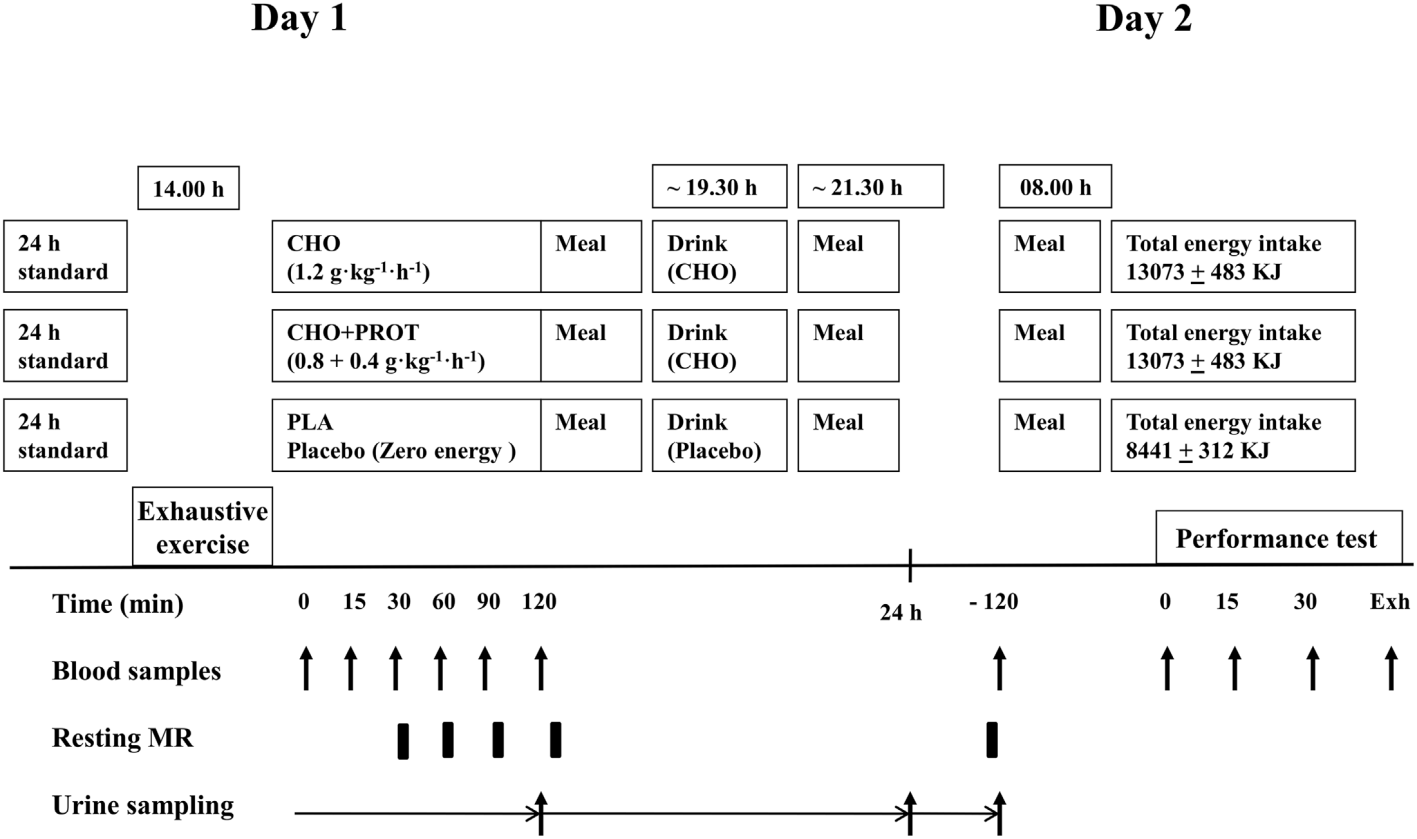
Schematic presentation of the study design. Abbreviations: Resting MR; Resting metabolic rate.

The subjects completed three performance tests after three different diet interventions supplied after bouts of exhaustive exercise. The study was double-blinded and randomized with a balanced crossover design. The interventions were separated by a minimum of 6 days. The protocol is described in Fig 1.

#### Initial tests

During the first day of the study, subjects performed an incremental test on a cycle ergometer (Lode Excalibur, Lode, Groningen; The Netherlands) at which time the relationship between work (Watt) and oxygen uptake (VO_2_) was established. Subjects started at 100 or 125 W (Mean 109.4 ± 6.6 W) and the load was increased by 25 Watt every 5 min. The final intensity was 237.5 ± 9.4 W. VO_2_ was measured on-line (Oxycon Pro; Jager Instruments, Hoechberg, Germany) over 1 min (from ~2.5–4 min at each intensity level) and capillary blood samples were taken for lactate analysis (1500 Sport, YSI Inc., Yellow Spring Instruments Ohio, USA). The incremental test was terminated when the lactate concentration had increased > 2 mM. The mean lactate concentration at the final intensity level was 2.4 ± 0.1 mM. Subjects selected a pedaling frequency between 85 and 95 rpm, and the self-selected pedaling frequency was used throughout the study. After 5 min of rest, maximal oxygen uptake (VO_2max_) was measured. Subjects started the VO_2max_ test at the highest (last) load of the incremental test and intensity was increased by 25 W every 30 s until voluntary exhaustion. Maximal lactate concentration averaged 8.2 ± 0.3 mM and RER was 1.11 ± 0.02 at the end of the VO_2max_ test. VO_2_ was measured over 30 s periods, and the mean of the two highest measurements was defined as maximal oxygen uptake (VO_2max_). The subjects returned to the laboratory 2 to 4 days after the test of VO_2max_ to complete a pretest in which subjects cycled 45 min at a load corresponding to ~72% of VO_2max_. This workload was calculated from the first incremental test (linear regression was used to establish the relationship between work and VO_2_). VO_2_ was measured between 3–5 min and the workload was adjusted to achieve a VO_2_ corresponding to ~72% of VO_2max_ (Watt_72%_). The defined load (Watt_72%_) was used in all tests at both day1 and day2.

### Diet interventions

Subjects recorded their dietary intake over the last 24 h prior to the first intervention period and were asked to follow the same diet prior to the two other interventions. No exercise was allowed for a minimum of 24 h before the exhaustive bout of exercise before the dietary interventions. Before the three diet intervention phases (Day1), subjects completed an exercise block with cycling until exhaustion. Subjects reported to the laboratory at 13.30 h and prepared for exhaustive bouts of exercise starting at 14.00 h (Fig 1). The training session was initiated with a 3 x 4 min warm up at 50, 55 and 60% of VO_2max_. Subjects then cycled at Watt_72%_ until exhaustion in 20 min intervals with 5 min rest between intervals. Exhaustion was defined as when subjects were unable to maintain the desired pedal frequency (self-selected pedal frequency ± 5 rpm) at the desired workload despite the third verbal encouragement to maintain the pedal frequency. After voluntary exhaustion, subjects were given 5 min of rest before completing a maximal number of 1 min intervals at 90% of VO_2max_ with 1 min rest between intervals. This protocol has been reported to reduce glycogen level in *m vastus lateralis* to a very low level [1]. VO_2_ and RER were measured after 4 min of the first interval and during the last 2 min of all intervals for calculation of carbohydrate oxidation. Capillary blood samples were taken before the first 20 min interval, immediately after each interval, at exhaustion and immediately after the last 1 min interval for measurements of lactate (described above) and glucose (HemoCue Glucose 201^+^, Ängholm, Sweden). Heart rate was measured continuously (Polar RS800CX, Kempele, Finland).

After the exhaustive exercise session, a catheter venflon was inserted into antecubital vein and a blood sample (7 ml) was taken. Subjects then received the first drink. For the first two hour of the diet interventions the subjects received a drink containing either: 1) 1.2 g carbohydrate (CHO) · kg^-1^ · h^-1^, 2) 0.8 g CHO · kg^-1^ · h^-1^ and 0.4 g whey · kg^-1^ · h^-1^ (CHO+PROT) or 3) similar flavored placebo without energy (PLA) in random order. The drink was supplied in equal portions every 30 min to obtain the energy level described. The CHO drink contained 85 g/L of glucose (Merck KGaA (Darmstadt, Germany) and 85 g/L maltodextrin (Carbo Flex Pure, Star Nutrition, Gymgrossisten Trollhättan, Sweden). CHO+PROT drink contained 56.5 g/L glucose and 56.5 g/L maltodextrin and 57 g/L whey isolate protein (Lacprodan, SP-9225 Instant, Arla, Aarhus, Danmark). PLA was water. All drinks contained 0.7 g/L NaCl and were flavored by 100 g/L Fun light (Stabburet, Norway). Drinks were served in opaque flasks and were indistinguishable by taste.

The first blood sample was taken before drink consumption, and capillary and venous blood samples were taken after15, 30, 60, 90 and 120 min of the recovery period as described above. Resting metabolic rate was measured after the blood samples were taken at 30, 60, 90 and 120 min of recovery according to the following protocol: subjects rested on a bed for 10 min before a V2 mask was used to collect air for 10 min.

After the first 2 h of the recovery period (and RER measurements) subjects had a standardized meal (composed of pasta, grained meat and tomato sauce). Energy content of the meal (and all other meals) was adjusted to body weight with macro nutrient content of: carbohydrate, 2.08 g · kg^-1^; protein, 0.59 g · kg^-1^; fat, 0.27 g· kg^-1^ (55.4 kJ ·kg^-1^). The subjects returned to their homes with additional drink and evening food. During the CHO and CHO+PROT trials, subjects received a drink containing 1.2 g CHO · kg^-1^ at 19.30 h, whereas PLA received flavored drink without energy. At 21.30 h, subjects had sandwiches with the following energy content: carbohydrate, 0.82 g · kg^-1^; protein, 0.27 g · kg^-1^; fat, 0.27 g· kg^-1^ (28.5 kJ ·kg^-1^). Water was allowed ad libitum. Subjects were not allowed to eat food other than that provided.

The following morning, subjects reported to the laboratory at 7.30 h and resting metabolic rate was measured. Subjects were not allowed to do any exercise for transportation to the laboratory, and rested 10 min in the bed before resting metabolic rate was measured as described. Fasted venous and capillary blood samples were taken and subjects received a light breakfast at 8 h. The breakfast had the following energy content: carbohydrate, 1.05 g · kg^-1^; protein, 0.17 g · kg^-1^; fat 0.18 g· kg^-1^ (27.4 Total kJ ·kg^-1^). During the diet intervention CHO and CHO+PROT were isocaloric (175 kJ · kg^-1^), but protein intake during the 18 h of recovery was 1.03 g · kg^-1^ and 1.83 g · kg^-1^ in CHO and CHO+PROT, respectively. The CHO and PLA interventions had the same intake of protein (1.03 g · kg^-1^) during the 18 h intervention, but carbohydrate intake was 7.55 g · kg^-1^ and 3.95 g · kg^-1^ in CHO and PLA, respectively. The study was designed with similar total energy intake in CHO and CHO+PRO during the 18 h recovery. Protein intake was higher in CHO+PROT (1.83 g · kg^-1^) than in CHO (1.03 g · kg^-1^) with carbohydrates substituted for protein in CHO (S1 Table). Protein intake was the same in CHO and PLA, but carbohydrate intake was reduced by 48% in PLA compared to CHO. Total energy intake during the 18 hour long recovery period was 13073 ± 483, 13073 ± 483 and 8441 ± 312 kJ in CHO, CHO+PROT and PLA, respectively.

### Performance test

The subjects reported to the laboratory at 7.30 h and resting metabolic rate was measured as described above. A fasting blood sample was taken before a standardized breakfast was served. A venflon was inserted into an antecubital vein at 9.30 h and subjects prepared for the performance test. The performance test was defined as cycling until exhaustion at Watt_72%_. The performance tests started approximately after 18 h of recovery and dietary intervention (Day2). Warm up started at 9.45 h and consisted of 3 x 4 min at 50, 55 and 60% of VO_2max_. The performance test started at 10.00 h and subjects cycled at Watt_72%_ (the same load as the day before corresponding to ~72% of VO_2max_) until exhaustion. Capillary and venous blood samples were taken before the performance test, after 15, 30 min, and every 30 min during the performance test, at exhaustion, and 15 and 30 min after exhaustion. VO_2_ and RER were measured after 4, 15, 30 min and thereafter every 30 min. VO_2_ and RER were also measured during the last min of exercise. Heart rate was recorded continuously during the performance test. Subjects reported rate of perceived exertion according to the Borg scale after 2, 15, 30 min and thereafter every 30 min. Subjects also reported rate of perceived exertion at exhaustion.

### Blood analyses

Venous blood samples (7 ml) were taken in tubes containing 140 μl 3 mM EGTA/2.5 mM glutathione in reduced form. Immediately thereafter, 2 ml of blood was transferred to another tube, which contained Aprotinin (500 KIE/ml) for measurement of glucagon and insulin. Blood samples were kept on ice until centrifugation (2500 g; 10 min; 4°C), and plasma was stored at -70°C until analysis. All analytic processes were conducted according to the manufacturer’s instructions.

#### Plasma glucose

Plasma glucose content was determined applying an enzymatic reference method GLUC assay (Roche Diagnostics GmbH, Mannheim, Germany) on a Cobas c111 system (Roche, Germany).

#### Insulin

Plasma insulin concentrations were measured with an enzyme-linked immunosorbent assay human insulin kit, K6219 (Dako, Glostrup, Denmark).

#### Glucagon

Plasma glucagon concentrations were determined by a radioimmunoassay (RIA) kit (EMD Millipore Corporation, Billerica, MA, USA).

#### Free Fatty Acids (FFA)

The FFA content was measured by an in vitro enzymatic colorimetric assay for the quantitative determination of non-esterified fatty acids (NEFA-HR) (Wako Chemicals GmbH, Neuss, Germany) using the autoanalyzer Cobas C-111 (Roche, Germany).

#### Glycerol

The glycerol content was measured by a direct colorimetric procedure (glycerol Randox, Crumlin, UK) using the autoanalyzer Cobas C-111 (Roche, Germany).

#### Creatine kinase

The Creatine Kinase (CK) content was measured by Siemens Dimension^®^ CKMB Creatine Kinase Isoenzyme MB assay, Creatine Kinase assay (Siemens, Newark, USA) and analyzed on a Dimension Vista 1500 (Siemens Healthcare GmbH, Erlangen, Germany).

#### Lactate Dehydrogenase

The content Lactate Dehydrogenase (LDH) was measured by a Lactate Dehydrogenase assay kit (Siemens, Newark, USA) and analyzed on a Dimension Vista 1500 (Siemens Healthcare GmbH, Erlangen, Germany).

#### Myoglobin

Myoglobin content was determined by immunoassay for the in vitro quantitative determination in human plasma, Myoglobin Assay (Roche Diagnostics GmbH, Mannheim, Germany), and analyzed on a Cobas 6000 E-modul (Roche Diagnostics GmbH, Germany).

#### Amino acids

For quantification of amino acids a targeted LC-MS/MS approach with iTRAQ^®^ (AA45/32^™^ Phys REAG Kit, Applied Biosystems, USA) labeling was used as described previously [35]. Plasma sample preparation was performed according to the manufacturer's instructions with 40 μl of sample volume.

#### Calculation of carbohydrate and fat oxidation

Carbohydrate (and fat) oxidation was calculated from RER and oxygen uptake according to Peronnet and Massicotte [36] as non-protein metabolism.

#### Urine urea and creatinine

Urine was collected during the recovery in three time periods (0–2 h; 2–8 h and 8–16 h) as shown in Fig 1. From the three periods, total volume was estimated and a 20 ml sample frozen at– 20°C for analysis of urea and creatinine. Urea and creatinine were determined on a MaxMat PL auto analyser (MaxMat PL, Montpellier, France) with a MaxMat urea kit (RM UREE0252) and a MaxMat creatinine PAP kit (RM CREP0270) according to instructions.

#### Calculation of nitrogen balance

Nitrogen balance was calculated from protein and nitrogen excretion over the 18 h recovery period. Nitrogen intake was calculated assuming the nitrogen to amino acid constant of 6.5 [37]. Nitrogen excretion was calculated from urea excretion as described by Rowlands et al. [29].

#### Motivation and rate of perceived exertion (RPE)

Motivation was rated on the 0–100 scale (from “no focus” to “perfect focus”) described previously [38]. RPE was rated on the 6–20 Borg scale [39].

#### Statistics

Data are presented as the mean±SEM. Comparisons of performance and nitrogen balance among treatments were performed with ANOVA and Fishers LSD was used as a post hoc test. Repeated measurements MANOVA were used to test responses in hormones, metabolites, and amino acids during the recovery period. Because time to exhaustion differed between subjects as well as diet, hormone, metabolites and amino acid responses at the 6 data points, blood samples (morning, before exercise, 15 min of exercise, exhaustion, and 15 min and 30 min after exercise) were evaluated by repeated measurements MANOVA. Variables are considered significant when p<0.05.

## Results

### Exhaustive exercise before the diet interventions (Day 1)

Subjects standardized their diet and exercise the last 24 hours before the bouts of exhaustive exercise prior to the dietary interventions (Day1); questionnaires confirmed adherence to instructions. Subjects cycled at 237 ± 6 Watt (Watt_72%_) and exhaustion occurred after 89.5 ± 10.1, 82.4 ± 7.8 and 87.3 ± 8.0 min prior to CHO, CHO+PROT and PLA, respectively. There was no difference in total cycling time before the three diet interventions. Mean oxygen uptake during the exhaustive exercise was 3.8 ± 0.1 L∙ min^-1^, which corresponded to 72.2 ± 1.1% of VO_2max_, during the exhaustive cycling at Watt_72%_. The participants completed 5 ± 1 1-min sprints at 90% of VO_2max_ after 5 min rest (after exhausting cycling); the number of sprints did not differ before the three interventions. Oxygen consumption increased slightly whereas RER decreased slightly during the exhaustive exercise at Watt_72%_ (Table 1). Total carbohydrate oxidation during the exhaustive cycling was 261 ± 28, 233 ± 22 and 226 ± 21 g before the CHO, CHO+PROT and PLA, respectively, and did not differ. Blood glucose decreased gradually during exercise and was 3.0 ± 0.1 mM at exhaustion (Table 1). Blood lactate rose from 0.73 ± 0.05 mM to about ~2.0 mM during the first 20 min interval and remained stable thereafter (Table 1). There were no differences in lactate responses during the exhaustive exercise prior to the three dietary interventions.

**Table 1:** Metabolic data during exhaustive exercise prior to the three diet interventions. Day 1.

	4 min	20 min	40 min	60 min	80 min	100 min	Exhaustion	After intervals
**Blood glucose (mM)**								
CHO	4.7 ± 0.1 (8)	4.3 ± 0.2 (8)* (8)*	3.5 ± 0.1 (8)*	3.2 ± 0.1 (6)	3.2 ± 0.2 (5)	3.0 ± 0.2 (4)	3.1 ± 0.2 (8)*	3.2 ± 0.1 (8)*
CHO+PROT	4.8 ± 0.2 (8)	3.8 ± 0.2 (8)*	3.3 ± 0.1 (8)*	3.1 ± 0.2 (7)	3.1 ± 0.1 (3)	3.0 ± 0.3 (2)	2.9 ± 0.1 (8)*	2.9 ± 0.1 (8)*†
PLA	4.7 ± 0.1 (8)	4.0 ± 0.1 (8)*	3.5 ± 0.2 (8)*	3.2 ± 0.2 (6)	3.2 ± 0.3 (5)	3.2 ± 0.0 (2)	2.9 ± 0.1 (8)*	3.0 ± 0.1 (8)*
**Blood lactate (mM)**								
CHO	0.76 ± 0.07 (8)	2.36 ± 0.35 (8)*	2.02 ± 0.34 (8)*	1.85 ± 0.35 (6)	1.66 ± 0.35 (5)	1.58 ± 0.35 (4)	2.00 ± 0.31 (8)*	3.09 ± 0.32 (8)*
CHO+PROT	0.74 ± 0.04 (8)	2.05 ± 0.24 (8)*	1.77 ± 0.23 (8)*	1.61 ± 0.21 (7)	1.64 ± 0.50 (3)	1.65 ± 0.70 (2)	1.86 ± 0.25 (8)*	2.92 ± 0.40 (8)*
PLA	0.70 ± 0.05 (8)	2.17 ± 0.30 (8)*	1.95 ± 0.27 (8)*	1.74 ± 0.23 (6)	1.67 ± 0.23 (5)	1.54 ± 0.55 (2)	1.96 ± 0.29 (8)*	3.28 ± 0.43 (8)*
**VO**_**2**_ **(l·min**^**-1**^**)**								
CHO	3.45 ± 0.09 (8)	3.66 ± 0.11 (8)*	3.74 ± 0.11 (8)*	3.67 ± 0.16 (6)	3.67 ± 0.14 (5)	3.74 ± 0.18 (4)	3.92 ± 0.11 (8)*	
CHO+PROT	3.43 ± 0.09 (8)	3.64 ± 0.08 (8)*	3.68 ± 0.09 (8)*	3.70 ± 0.11 (7)	3.96 ± 0.13 (3)	4.04 ± 0.08 (2)	3.85 ± 0.11 (8)*†	
PLA	3.47 ± 0.07 (8)	3.66 ± 0.09 (8)*	3.76 ± 0.09 (8)*	3.72 ± 0.11 (6)	3.81 ± 0.15 (5)	4.16 ± 0.08 (2)	3.96 ± 0.13 (8)*	
**RER (VCO**_**2**_**/VO**_**2**_**)**								
CHO	0.94 ± 0.01 (8)	0.90 ± 0.01 (8)*	0.89 ± 0.01 (8)*	0.88 ± 0.01 (6)	0.86 ± 0.01 (5)	0.84 ± 0.01 (4)	0.84 ± 0.02 (8)*	
CHO+PROT	0.94 ± 0.01 (8)	0.89 ± 0.01 (8)*	0.88 ± 0.01 (8)*	0.87 ± 0.02 (7)	0.83 ± 0.03 (3)	0.82 ± 0.05 (2)	0.85 ± 0.02 (8)*	
PLA	0.93 ± 0.01 (8)	0.88 ± 0.01 (8)*†	0.87 ± 0.01 (8)*	0.88 ± 0.01 (6)	0.86 ± 0.01 (5)	0.84 ± 0.01 (2)	0.83 ± 0.02 (8)*	
**CHO oxidation (g·min**^**-1**^**)**								
CHO	3.6 ± 0.2 (8)	3.2 ± 0.2 (8)*	3.0 ± 0.2 (8)*	2.9 ± 0.2 (6)	2.4 ± 0.2 (5)	2.2 ± 0.3 (4)	2.3 ± 0.3 (8)*	
CHO+PROT	3.5 ± 0.2 (8)	3.0 ± 0.1 (8)	2.9 ± 0.2 (8)*	2.7 ± 0.3 (7)	2.0 ± 0.5 (3)	1.9 ± 0.8 (2)	2.4 ± 0.4 (8)*	
PLA	3.4 ± 0.2 (8)	2.8 ± 0.1 (8)*†	2.6 ± 0.1 (8)*	2.4 ± 0.3 (6)	2.2 ± 0.2 (5)	2.0 ± 0.8 (2)	2.2 ± 0.3 (8)*	
**HF (beat·min**^**-1**^**)**								
CHO	146 ± 5 (8)	153 ± 4 (8)*	154 ± 4 (8)*	155 ± 5 (6)	156 ± 5 (5)	163 ± 7 (4)	162 ± 4 (8)*	
CHO+PROT	147 ± 5 (8)	153 ± 5 (8)*	155 ± 5 (8)*	158 ± 5 (7)	152 ± 6 (3)	161 ± 7 (2)	163 ± 4 (8)*	
PLA	146 ± 5 (8)	154 ± 4 (8)*	157 ± 4 (8)*	159 ± 6 (6)	161 ± 7 (5)	164 ± 0 (2)	165 ± 5 (8)*	

Values are means ± SEM. Time to exhaustion varied between subjects; number of subjects at different time points is given in parentheses. RER: Respiratory exchange ratio; CHO: Carbohydrate; HF: Heart frequency.

* Significantly different from values at 4 min (p<0.01).

^†^ Significantly different from CHO (p<0.05).

### Recovery period

**Fig 2:**
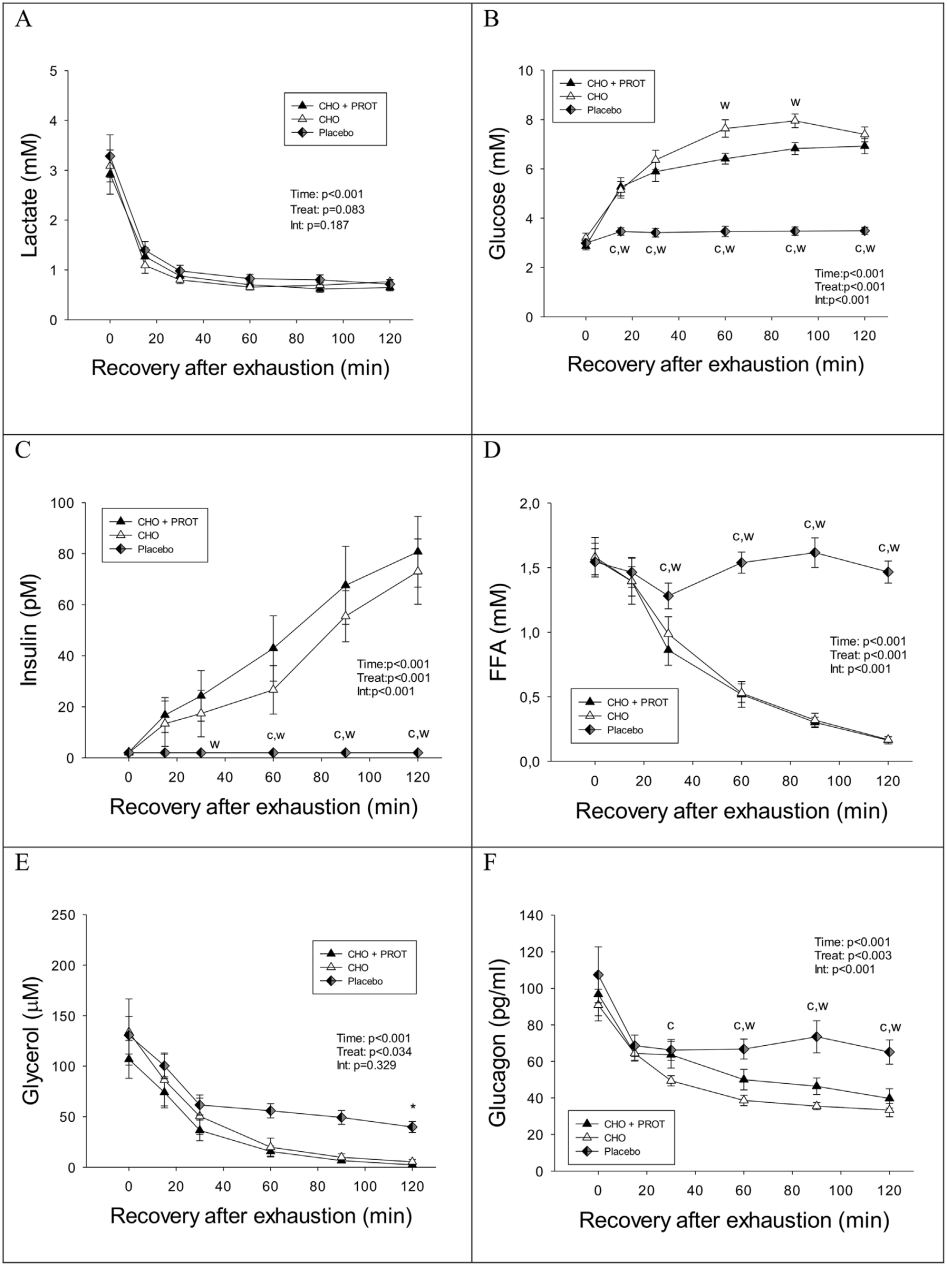
Plasma concentrations of lactate, glucose, insulin, FFA, glycerol and glucagon during the first 2 h recovery when provided CHO, CHO+PROT or PLA. Data are mean and error bars represents SEM. C: p<0.05 compared to CHO; W: p<0.05 compared to CHO+PROT (whey); P: p<0.05 compared to placebo. N = 8 for all data points.

Blood lactate concentrations decreased rapidly after exhaustive exercise independent of dietary intervention. Lactate levels declined rapidly and were back at baseline after 30 minutes in all three dietary interventions (Fig 2A). Blood glucose concentration increased rapidly after intake of CHO and CHO+PROT (Fig 2B). At 60 and 90 min the blood glucose concentration was significantly higher after intake of CHO compared to CHO+PROT (Fig 2A). In PLA blood glucose remained low (~3.5 mM) during the 2 h of recovery after the exhaustive bout of exercise and was significantly lower than CHO and CHO+PROT. Insulin concentration rose after ingestion of CHO and CHO+PROT, but not in PLA (Fig 2C). There was a tendency for a higher insulin concentration at 60 minutes (p = 0.053) after ingestion of CHO+PROT as compared to CHO alone. Area under curve also tended to be higher for insulin after CHO+PROT compared to CHO (p<0.06). FFA concentration decreased rapidly after intake of CHO and CHO+PROT, but remained elevated after PLA (Fig 2D). Plasma glycerol decreased rapidly after exhaustive exercise but was higher in PLA than CHO and CHO+PROT in the 60–120 min period (Fig 2E). Glucagon concentration decreased after exhaustive exercise, but more so in CHO and CHO+PROT than in PLA (Fig 2F).

VO_2_ and RER where determined every 30 min during the first 2 h of recovery and used to calculate carbohydrate oxidation rate (S2 Table). RER values were suppressed below 0.70 during the first 30 min of recovery in each of the treatments. RER increased during intake of CHO and CHO+PROT, but RER remained below 0.70 in PLA during the first 2 h of recovery. VO_2_ was higher after intake of CHO+PROT as compared to CHO and PLA.

**Fig 3:**
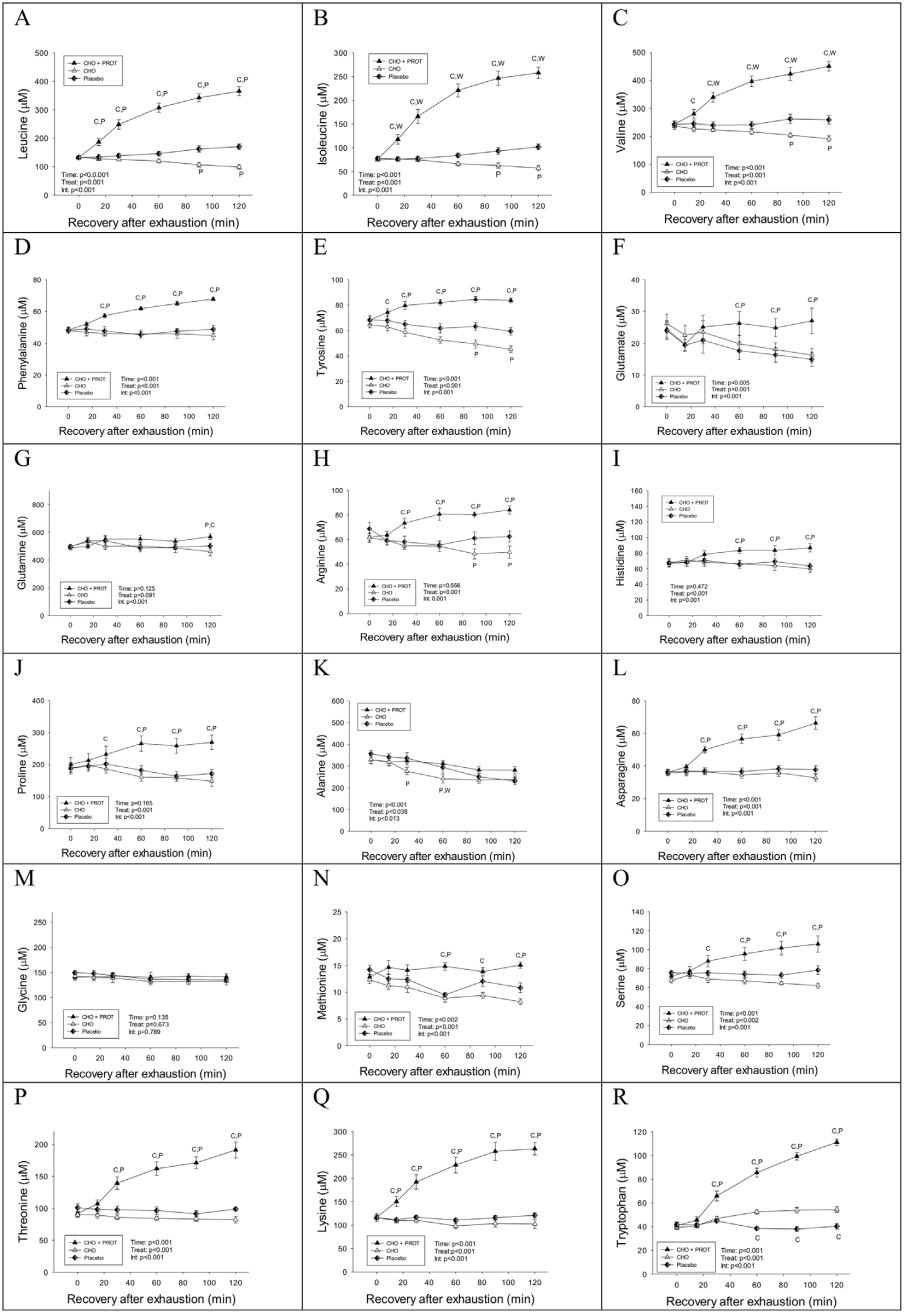
Plasma concentrations of amino acids during the first 2 h of recovery when provided CHO, CHO+PROT or PLA. Data for 18 amino acids are presented; no data are presented on cysteine and aspartate. Data are mean and error bars represents SEM. C: p<0.05 compared to CHO; W: p<0.05 compared to CHO+PROT (whey); P: p<0.05 compared to placebo. N = 8 for all data points.

Concentrations of amino acids during the three interventions are shown in Fig 3. Plasma concentrations of many amino acids including BCAA increased after intake of CHO+PROT whereas their concentrations remained unchanged in PLA (Fig 3A–3C). In contrast, the concentrations of BCAA decreased during the 2 h CHO supplementation, most likely due to increased BCAA clearance resulting from an increase in plasma insulin. The concentration of the aromatic amino acids (phenylalanine and tyrosine), which are essentially not degraded in skeletal muscle, remained stable in PLA. In CHO+PROT, concentrations of several amino acids increased (arginine, proline, asparagine, serine, threonine, lysine and tryptophan) while remaining stable during PLA (Fig 3). Amino acid concentrations at exhaustion did not differ among exhaustive exercises prior to the three dietary interventions. Mean concentrations of plasma amino acids at exhaustion are shown in S3 Table.

**Fig 4:**
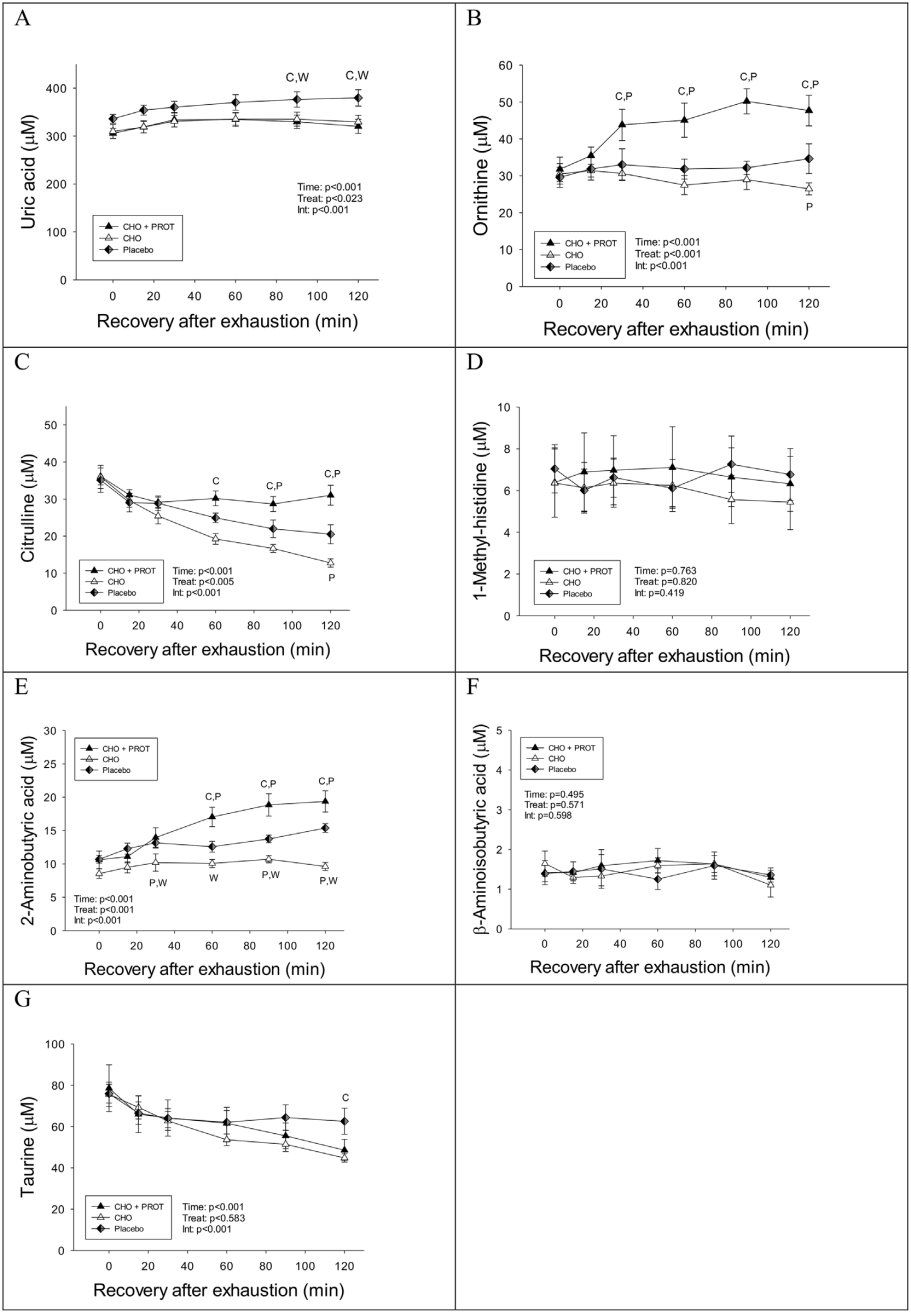
Plasma concentrations of uric acid, ornithine, citrulline and 1-methyl-histidine during the first 2 h of recovery when provided CHO, CHO+PROT or PLA. Data are mean and error bars represents SEM. C: p<0.05 compared to CHO; W: p<0.05 compared to CHO+PROT (whey); P: p<0.05 compared to placebo. N = 8 for all data points.

Uric acid increased gradually during the recovery period and was higher in PLA compared to CHO and CHO+PROT after 60 min (Fig 4A). Concentrations of ornithine increased in CHO+PROT, but remained stable in CHO and PLA (Fig 4B). Data on other amino and organic acids are shown in Fig 4. Citrulline remained stable during intake of CHO+PROT, but decreased in CHO and PLA, and more in CHO than in PLA. Dietary intervention had no effect of plasma 1-methyl-histidine during the first 2 h of recovery.

### Fasted values the following morning (Day 2)

Fasting blood glucose levels were significantly higher after ingestion of CHO and CHO+PROT compared to PLA (p<0.05; Table 2) the morning after the initial exhaustive exercise. RER and CHO oxidation was significantly higher in CHO compared to PLA (p<0.05), but there was no difference between CHO and CHO+PROT. Fasting insulin, FFA, glycerol and glucagon levels did not differ between diet interventions.

**Table 2:** Fasting blood glucose, lactate, VO_2_, RER, carbohydrate oxidation and HR after the three interventions.

	CHO	CHO+PRO	PLA
**Blood glucose (mM)**	4.5 ± 0.1*	4.5 ± 0.1*	4.2 ± 0.1
**Blood lactate (mM)**	0.54 ± 0.04	0.50 ± 0.05	0.44 ± 0.03
**VO**_**2**_ **(l·min**^**-1**^**)**	0.26 ± 0.01	0.27 ± 0.01	0.26 ± 0.02
**RER (VCO**_**2**_**/VO**_**2**_**)**	0.78 ± 0.01*	0.76 ± 0.01	0.75 ± 0.01
**CHO oxidation (g·min**^**-1**^**)**	0.09 ± 0.01*	0.07 ± 0.01	0.05 ± 0.01
**HF (beat·min**^**-1**^**)**	56 ± 3	60 ± 3	57 ± 2

Values are means ± SEM. n = 8. The measurements are done in the morning at 08.00 AM after overnight fast. CHO ox: Carbohydrate oxidation.

* Significantly different from PLA (p<0.05).

Fasting concentrations of most amino acids (asparagine, glutamine, glycine, histidine, isoleucine, leucine, lysine, phenylalanine, proline, serine, threonine, tryptophan) were increased similarly in CHO+PROT, CHO and PLA compared to values immediately after exhaustive exercise the day before (S3 Table). Fasting morning concentrations of lysine, proline and threonine were higher after CHO+PROT compared to both CHO and PLA. Concentration of valine increased only in CHO+PROT and PLA, and was lower in CHO compared to CHO+PROT and PLA. Concentration of alanine decreased significantly compared to immediately after exhaustion Day 1, with no difference among dietary interventions. Plasma organic acids are shown in S4 Table.

### Time to exhaustion (TTE) at the performance tests the day after the dietary interventions

**Fig 5:**
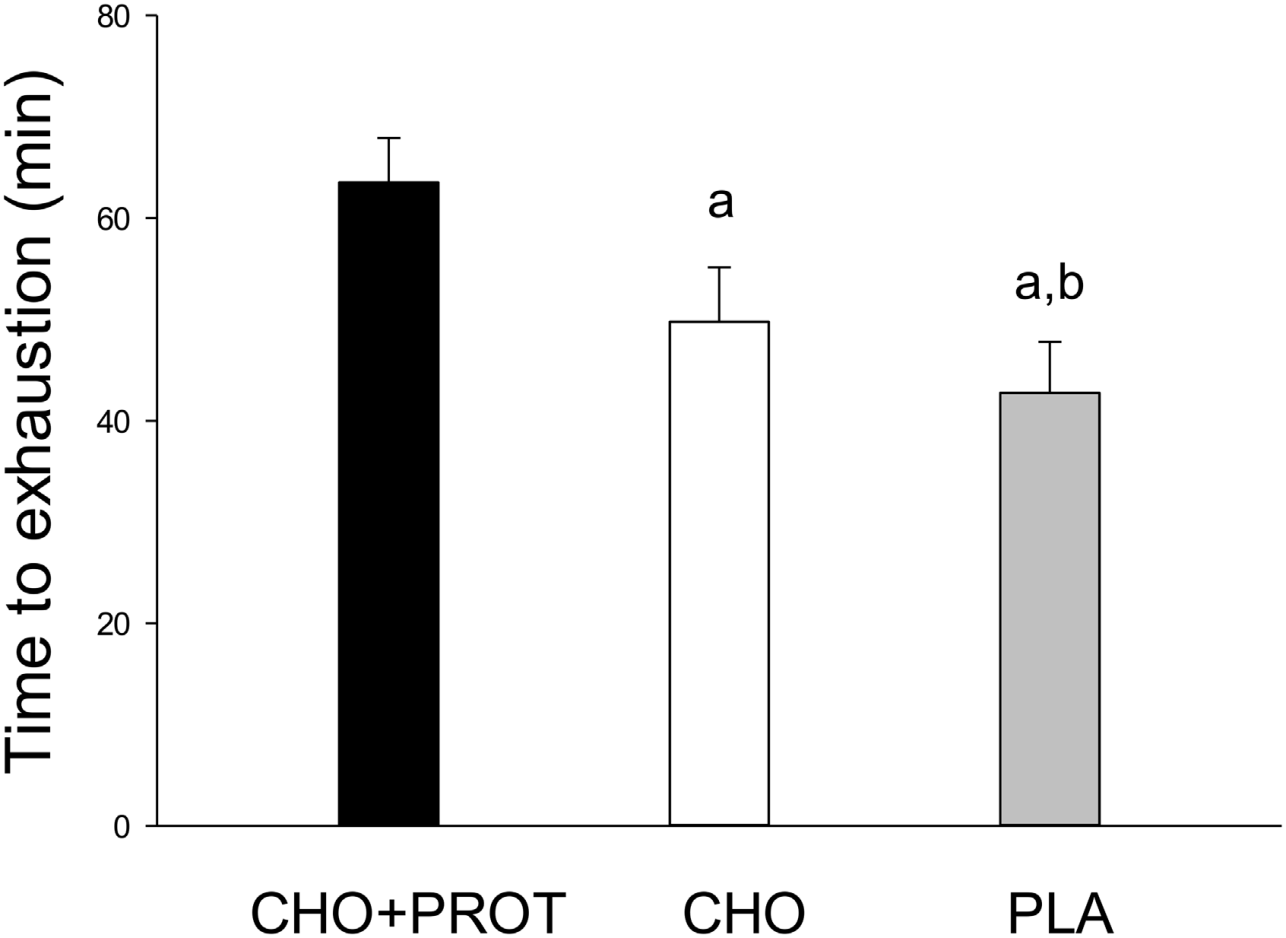
Performance test after 18 h recovery receiving CHO, CHO+PROT or PLA. The performance test was time to exhaustion cycling at W_72%_ (237±6 W). Data are mean and error bars represents SEM. N = 8 at all tests. a: p<0.05 compared to CHO+PROT; b: p<0.05 compared to CHO.

For the performance test after the interventions (Day2) time to exhaustion cycling at Watt_72%_ (237 ± 6 W) was longer after CHO+PROT compared to CHO (63.5 ± 4.4 versus 49.8 ± 5.4 min; p<0.05) (Fig 5). For PLA, time to exhaustion was 42.8 ± 5.1 min, which was lower than CHO (p<0.05). Noteworthy is our observation that cycle time was longer in all test subjects after ingestion of CHO+PROT compared to CHO and PLA. However, prior to the dietary interventions (Day1) participants also cycled to exhaustion at Watt_72%_ (237 ± 6 W), but in intervals of 20 min with 5 min rest between intervals.

### Metabolic data during performance tests (TTE) the day after the dietary interventions (Day2)

Oxygen uptake during the TTE (237 ± 6 W) was similar after all diet interventions and the oxygen uptake during Day2 corresponded to 73.2 ± 0.9% of VO_2max_. RER and carbohydrate oxidation during the TTE were significantly lower in PLA after 4 and 15 min compared to CHO (p<0.01; Table 3). There was no difference between CHO and CHO+PROT at the start of TTE. After 15 min, RER and carbohydrate oxidation were significantly lower after ingestion of PLA compared with CHO and CHO+PROT (p<0.01). Total carbohydrate oxidation during TTE was 136 ± 14, 157 ± 13 and 96 ± 11 g after ingestion of CHO, CHO+PROT and PLA, respectively (Table 3). Total carbohydrate oxidation was significantly higher after ingestion of CHO+PROT compared to CHO (p<0.05) and PLA (p<0.01) and CHO compared to PLA (p<0.01).

**Table 3:** VO_2_, RER, CHO oxidation and HF during the performance test (TTE) after the three diet interventions (Day 2).

	4 min	15 min	30 min	60 min	Exhaustion
**VO**_**2**_ **(l·min**^**-1**^**)**					
CHO	3.56 ± 0.09 (8)	3.74 ± 0.10 (8)*	3.92 ± 0.06 (7)	4.09 ± 0.05 (2)	3.93 ± 0.15 (8)*
CHO+PROT	3.53 ± 0.09 (8)	3.78 ± 0.06 (8)*	3.86 ± 0.08 (8)	3.94 ± 0.12 (3)	3.96 ± 0.05 (8)*
PLA	3.61 ± 0.07 (8)	3.83 ± 0.09 (8)*,^C^	3.96 ± 0.12 (5)	-	3.94 ± 0.11 (8)*
**RER (VCO**_**2**_**/VO**_**2**_**)**					
CHO	0.93 ± 0.01 (8)	0.88 ± 0.01 (8)*	0.86 ± 0.01 (7)	0.82 ± 0.01 (2)	0.85 ± 0.01 (8)*
CHO+PROT	0.91 ± 0.01 (8)	0.87 ± 0.01 (8)*	0.86 ± 0.01 (8)	0.83 ± 0.01 (3)	0.83 ± 0.01 (8)*
PLA	0.89 ± 0.02 (8) ^C^	0.83 ± 0.01 (8)*,^C^,^W^	0.82 ± 0.02 (5)	-	0.83 ± 0.01 (8)*
**CHO oxidation (g·min**^**-1**^**)**					
CHO	3.4 ± 0.2 (8)	2.8 ± 0.2 (8)*	2.6 ± 0.2 (7)	2.1 ± 0.2 (2)	2.5 ± 0.2 (8)*
CHO+PROT	3.2 ± 0.2 (8)	2.7 ± 0.2 (8)*	2.4 ± 0.2 (8)	2.1 ± 0.2 (3)	2.2 ± 0.2 (8)*
PLA	3.0 ± 0.3 (8) ^C^	2.2 ± 0.2 (8)*,^C^,^W^	2.0 ± 0.3 (5)	-	2.1 ± 0.2 (8)*
**HF (beat·min**^**-1**^**)**					
CHO	151 ± 4 (8)	157 ± 3 (8)*	163 ± 2 (7)	165 ± 3 (2)	164 ± 3 (8)*
CHO+PROT	151 ± 4 (8)	157 ± 3 (8)*	161 ± 3 (8)	167 ± 4 (3)	166 ± 3 (8)*
PLA	154 ± 4 (8)	162 ± 3 (8)*,^C^,^W^	169 ± 2 (5)	-	169 ± 4 (8)*,^C^,^W^

Values are means ± SEM. () number of subjects.

* Significantly different from values at 4 min (start of the time to exhaustion test) (p<0.01).

^C^: Significantly different form CHO (p≤0.01).

^W^: Significantly different from CHO+PROT (p<0.05).

**Fig 6:**
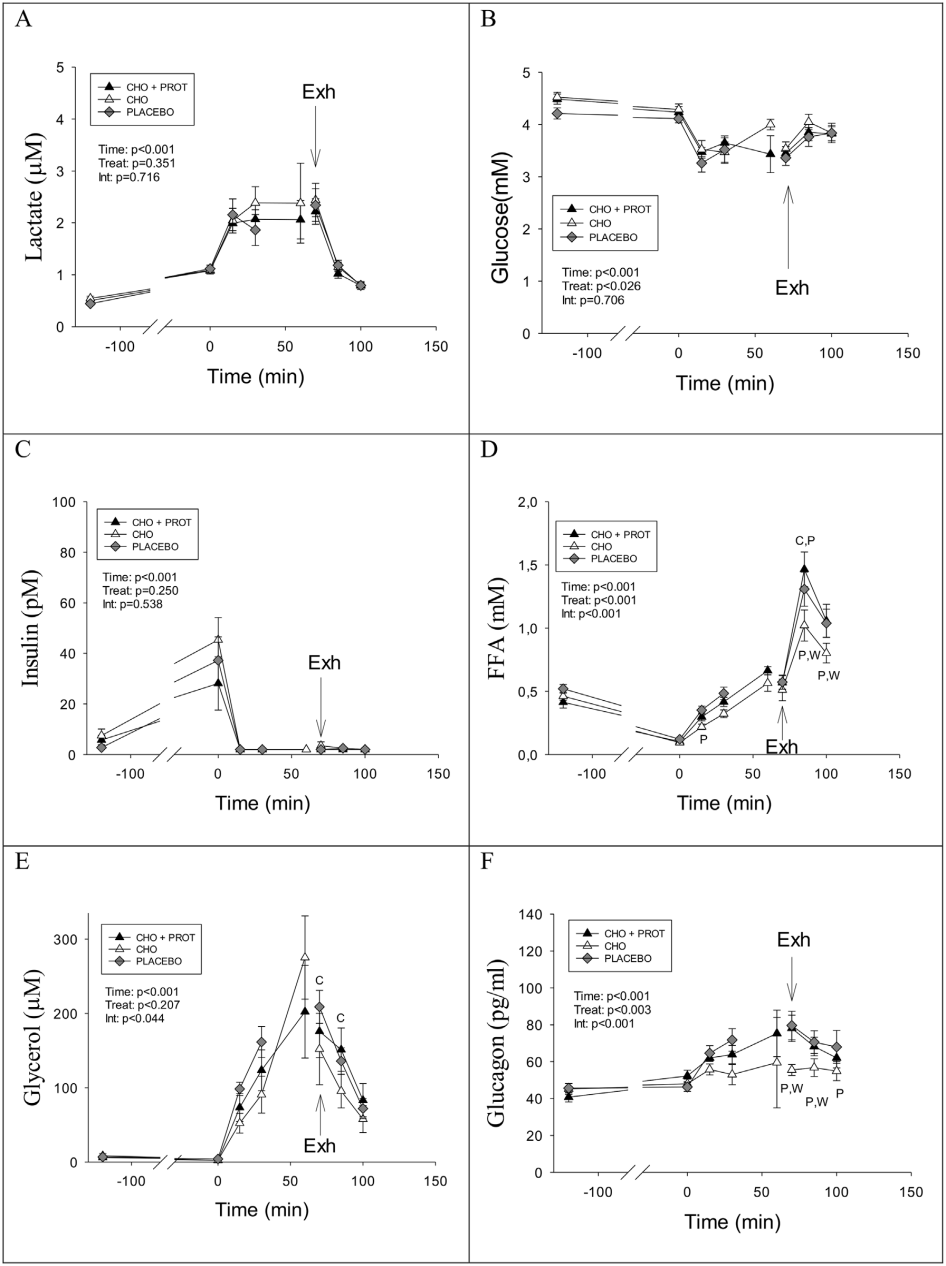
Concentrations of metabolites and hormones in fasted condition the day after exhaustive exercise and diet interventions, and during and after the performance test. Data are mean and error bars represents SEM. C: p<0.05 compared to CHO; W: p<0.05 compared to CHO+PROT (whey); P: p<0.05 compared to placebo. N = 8 for all data points.

Lactate increased during the TTE and there were no differences at any time point after the three dietary interventions (Fig 6A). Fasting glucose was lower in PLA compared to CHO (Fig 6B). During TTE the blood glucose concentration declined significantly in each treatment. The blood glucose concentration was significantly lower after 15 min of cycling after ingestion of PLA compared to CHO and CHO+PROT (p<0.05; ANOVA comparison at time point). There were no differences in glucose concentration at exhaustion among treatments. Fasted plasma insulin was low and similar between diet interventions, and increased similarly after breakfast on Day2 (Fig 6C). During the TTE, plasma insulin dropped to low levels in all treatments.

The plasma FFA responses during the TTE were different among the diet interventions. Interestingly, plasma FFA was lower in CHO compared to PLA after 15 min of exercise (Fig 6D). Furthermore, after the TTE, plasma FFA increased to higher levels in CHO+PROT and PLA compared to CHO. Plasma glycerol also responded differently during TTE after the dietary interventions (Fig 6E), and suggested that lipolysis was reduced in CHO compared with CHO+PROT. Plasma glucagon responses during the TTE differed after the dietary interventions, and the results may be of importance for understanding the lower time to exhaustion in CHO. Interestingly, glucagon did not increase significantly during the TTE in CHO (Fig 6F) and was higher in PLA and CHO+PROT at exhaustion.

During TTE oxygen uptake increased with all dietary interventions, but the increase was faster in PLA compared to CHO (Table 3). RER (and carbohydrate oxidation) decreased gradually during the TTE with RER being lower for PLA compared to CHO and CHO+PROT after 15 min of exercise (Table 3). Heart rate increased gradually after all interventions, but was higher at exhaustion in PLA compared to CHO and CHO+PROT (Table 3).

### Amino acids

**Fig 7:**
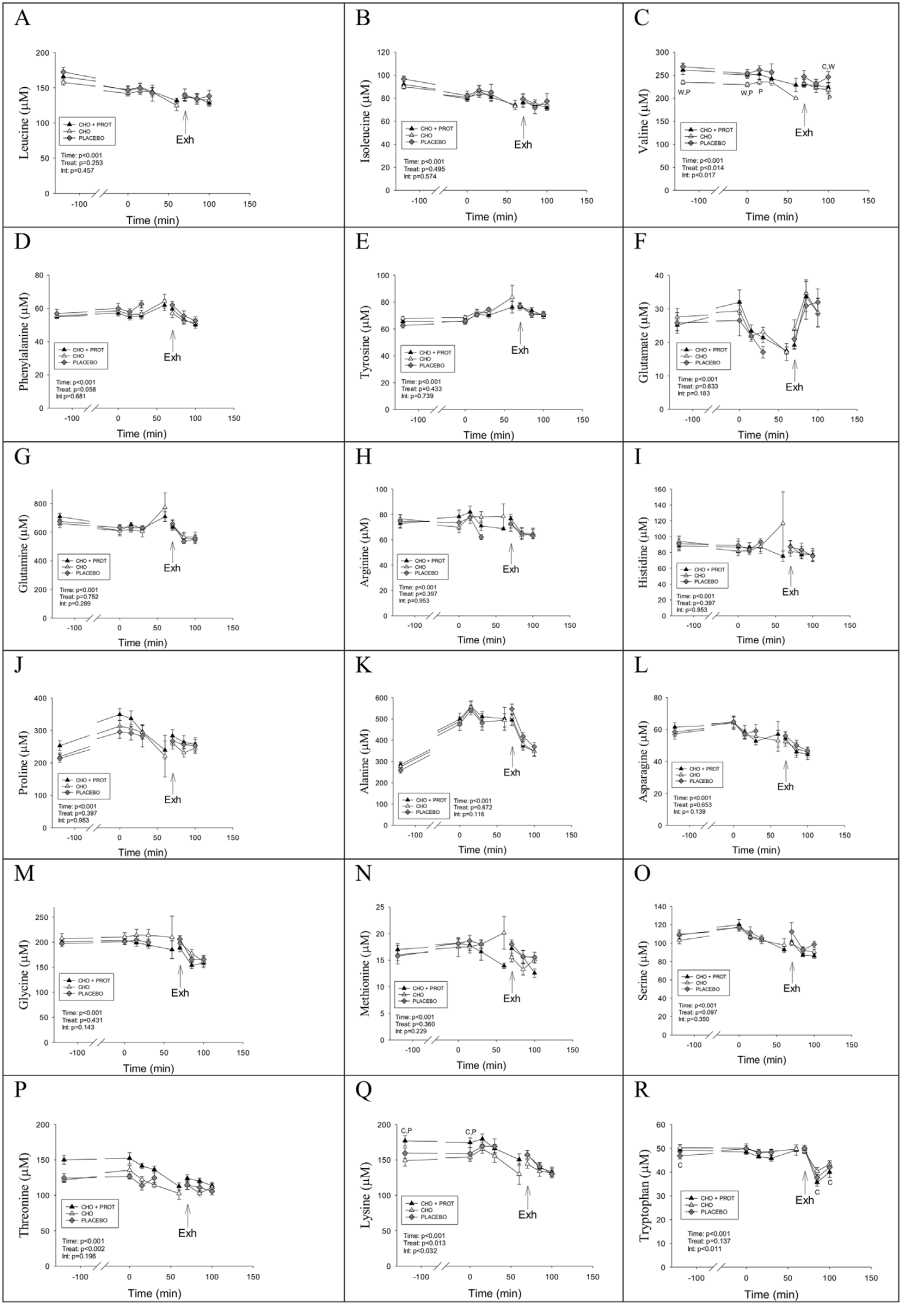
Concentrations of amino acids in fasted condition the day after exhaustive exercise and diet interventions, and during and after the performance test. Data are mean and error bars represents SEM. C: p<0.05 compared to CHO; W: p<0.05 compared to CHO+PROT (whey); P: p<0.05 compared to placebo. N = 8 for all data points.

The concentrations on most amino acids were similar before the TTE among treatments (Fig 7). However, the concentration of valine was lower in CHO compared to CHO+PROT and PLA (Fig 7C), whereas the concentrations of threonine and lysine were higher after CHO+PROT compared to CHO and PLA (Fig 7P and 7Q). Concentration of tryptophan was lower in PLA compared to CHO after exercise. During the TTE all amino acids responded similarly among treatments except valine, which remained lower in CHO compared to PLA (Fig 7C). Interestingly, glutamate decreased significantly during TTE under all dietary interventions (Fig 7F). There were no differences in responses in ornithine and citrulline during the TTE after the three dietary interventions (S1 Fig). Taurine increased during the TTE after all dietary interventions (S1 Fig).

### Nitrogen balance

**Fig 8:**
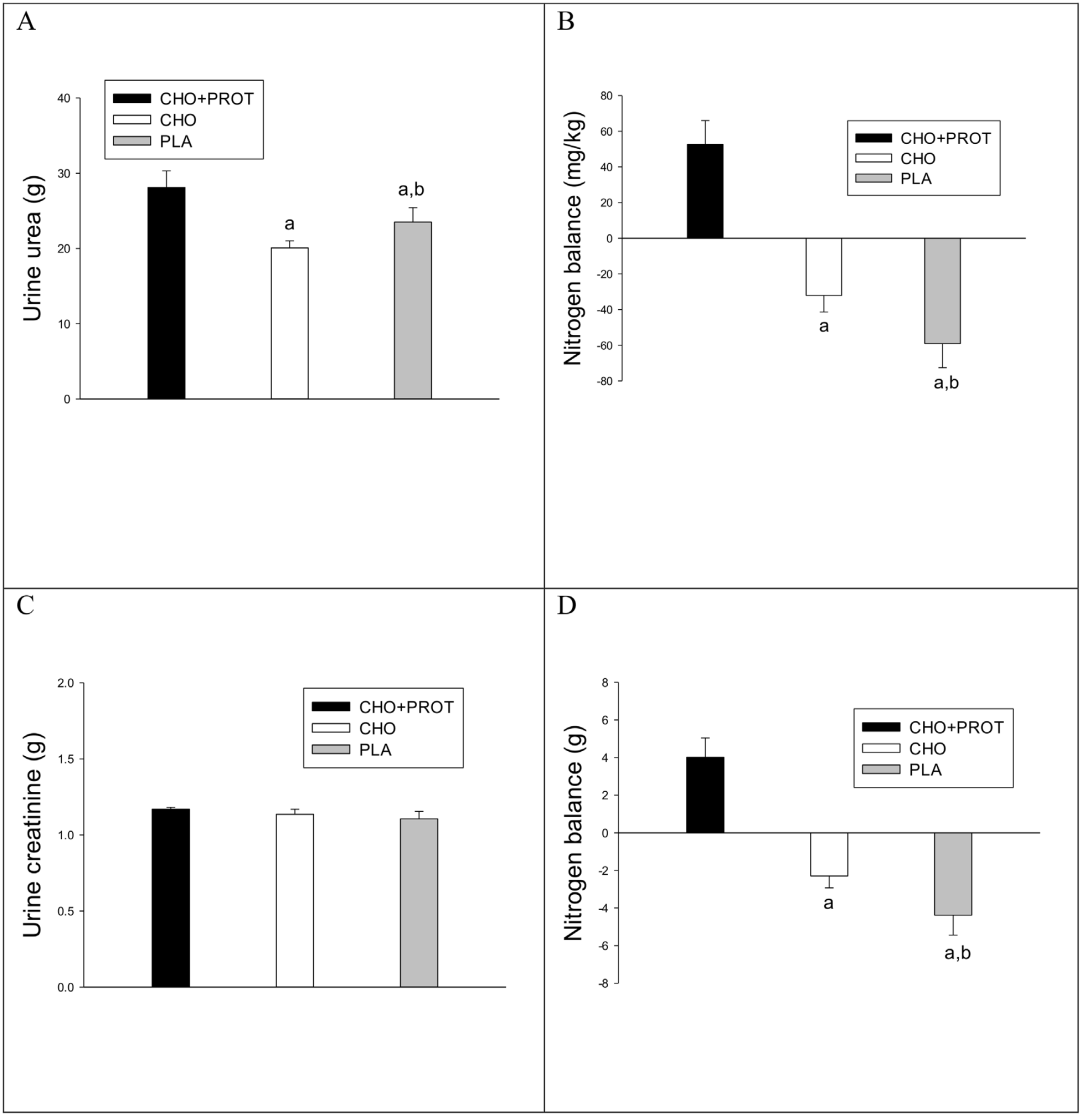
Nitrogen balance and urine excretion of urea and creatinine. a: p<0.05 compared to CHO+PROT; b: p<0.05 compared to CHO. Data are mean and error bars represents SEM. C: p<0.05 compared to CHO; W: p<0.05 compared to CHO+PROT (whey); P: p<0.05 compared to placebo. N = 8 for all data points.

Urine sampling was initiated at the end of the exhaustive exercise prior to the dietary intervention (Day 1) until the following morning; the day of the performance test. Total nitrogen excretion was higher in CHO+PROT compared to CHO and PLA (Fig 8A). Nitrogen balance was positive in CHO+PROT and negative in CHO and PLA (Fig 8B) with nitrogen balance more negative in PLA compared to CHO (Fig 8B). Creatinine excretion was similar during the three diet interventions (Fig 8C). Plasma uric acid remained higher after PLA the following morning and remained higher than CHO and CHO-PROT during the TTE (S1 Fig).

### Muscle damage

Plasma CK, LDH and myoglobin were measured as markers of muscle damage. The dietary interventions did not influence the markers for muscle damage after exhaustive cycling (S5 Table).

### Motivation and rate of perceived exertion

**Fig 9:**
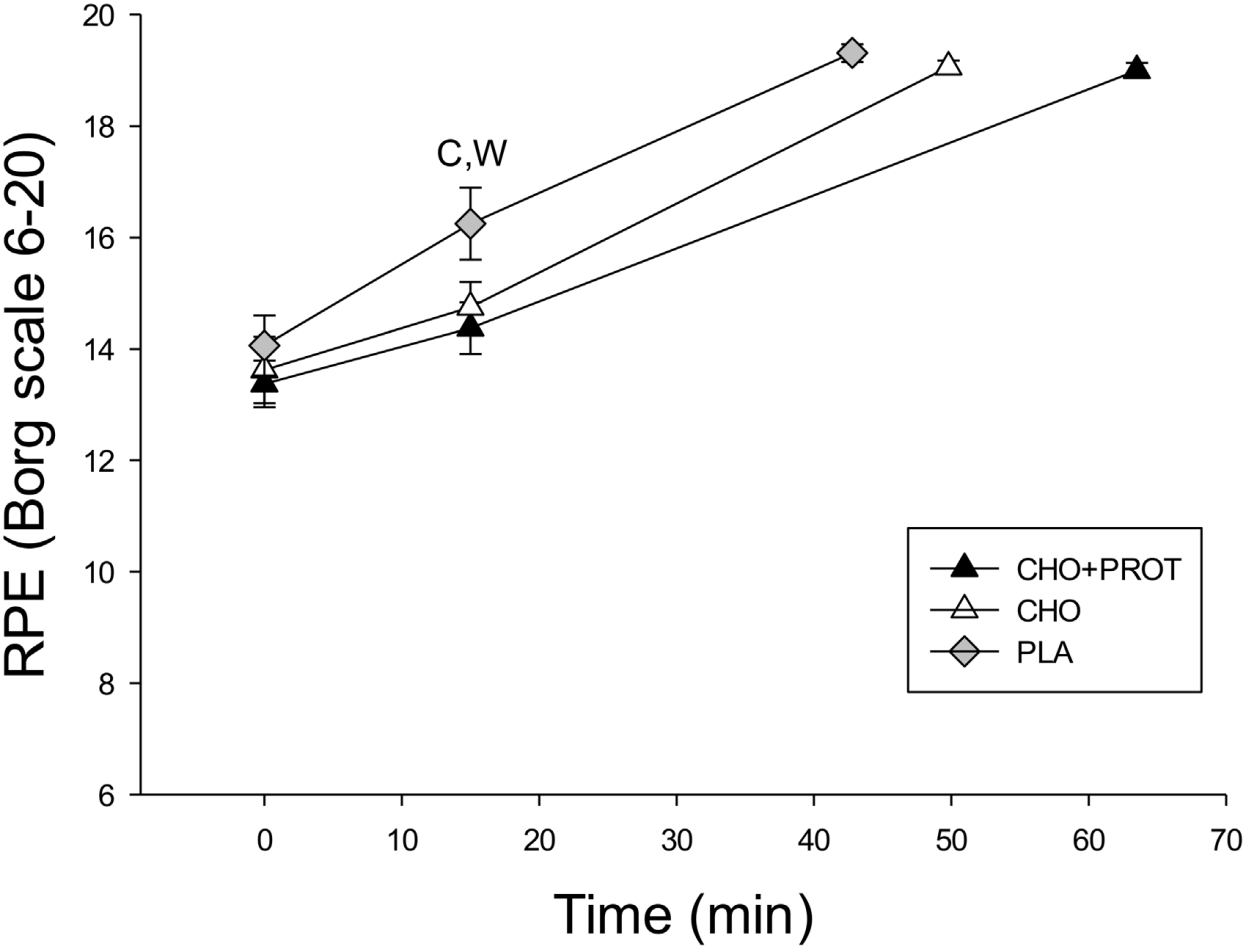
Rate of perceived exertion (RPE) during the time to exhaustion (TTE) performance test after the three diet interventions. Data are mean and error bars represents SEM. C: p<0.05 compared to CHO; W: p<0.05 compared to CHO+PROT (whey); P: p<0.05 compared to placebo. N = 8 for all data points.

On a scale from 0–100 the motivation to perform during TTE was 76 ± 4, 80 ± 3 and 84 ± 3 after ingestion of CHO, CHO+PROT and PLA. These numbers correspond to high motivation to perform maximally, and there were no differences in motivation among the three dietary interventions. There also were no differences in rating of perceived exertion among the three dietary interventions at the beginning of the TTE (Fig 9). However, at 15 min of cycling rating of perceived exertion was significantly higher after ingestion of PLA compared to CHO+PROT and CHO (p<0.05). RPE at exhaustion was similar during the three performance tests (TTE).

## Discussion

The main finding in the present study is that a CHO+PROT supplementation during the first 2 h after exhaustive cycling was superior to an isocaloric carbohydrate supplement for cycling performance recover when assessed 18 h later. CHO+PROT supplementation immediately after exercise increased plasma concentrations of glucose, insulin, BCAA and a number of other amino acids. CHO supplementation also caused increases in plasma glucose and insulin, but the concentration of many amino acids decreased, probably caused by insulin effects on amino acid removal from plasma. Nitrogen balance during the recovery period was positive after intake of protein immediately after exhaustion (CHO+PROT), but was negative in CHO despite that 1.03 g protein ∙ kg body weight^-1^ was provided during the 18 h recovery. Reducing the intake of carbohydrates (PLA) during the recovery period resulted in a more negative protein balance and reduced recovery of performance compared to CHO. Our results suggest that CHO+PROT during the first 2 h of an 18 h recovery period accelerates repair of protein function and possibly resynthesis of muscle glycogen stores [6,23–25] resulting in improved exercise performance at the end of recovery. Limiting carbohydrate intake during recovery also adversely affected recovery, and this may be related to the inability to recover sufficient muscle glycogen stores [34].

In the present study cycling time to exhaustion after 18 h of recovery was longer after CHO+PROT supplementation compared to CHO, when the same workload (Watt_72%_) was used to induce exhaustion prior to dietary intervention and during the performance test the following day. The effect of CHO+PROT intake during the first 2 h after exhaustive exercise was convincing as all 8 subjects performed best after intake of CHO+PROT compared to both CHO and PLA. The findings of improved performance after ingestion of protein combined with carbohydrates immediately after exhaustion as compared to carbohydrates only are in line with those of several other studies using time to exhaustion as the performance test [21,26]. Saunders et al. also showed a dramatic effect of protein intake when subjects cycled to exhaustion at 75% VO_2max_ and 16 h later used cycling to exhaustion at 85% VO_2max_ to test recovery of performance [26]. Moreover, Ferguson-Stegall et al. found a significant increase in cycling time trial performance after 4 h of exercise recovery when a CHO+PRO supplement was provided as compared with an isocaloric CHO supplement or placebo [20]. However, co-ingestion of protein with carbohydrate after exercise has not always been found to have a positive effect on recovery of performance [31,40]. Furthermore, Rowlands et al. found a positive effect of protein supplementation post exercise on recovery of performance in some [28,29] but not in other studies [32,41]. Berardi et al. also reported improved performance after CHO+PROT compared to CHO in one [27] but not in another study [24]. Several reasons may explain the differences, which may include the type of exercise before the recovery period, the type of post-recovery performance test, the duration of the recovery period, and the amount and type of protein consumed.

The initial glycogen content is a determining factor for time to exhaustion at intensities of ~70% of VO_2max_ [5], and protein intake in combination with carbohydrate during the early phase of recovery has been shown in some studies to produce a higher rate of glycogen synthesis compared with carbohydrate intake alone [6,23,24]. In the present study, muscle glycogen was not measured, but plasma insulin tended to be higher in CHO+PROT compared to CHO whereas glucose concentration was higher in CHO compared to CHO+PROT during the first 2 hours of recovery suggesting a greater rate of glucose clearance and possibly glycogen synthesis during the CHO+PROT for this time period [23]. However, there are no data supporting higher glycogen content 18 h after exhaustive exercise when fed CHO+PROT compared to isocaloric CHO. Resting RER normally correlates with glycogen stores [42] and resting RER was higher in CHO than PLA as expected [43], and resting RER was similar in CHO and CHO+PROT groups suggesting similar glycogen content. Assuming a glycogenolytic rate of ~1.0 mmol·kg^-1^·min^-1^ during cycling at ~ 72% VO_2max_ [1,2] the 14 min longer cycle time achieved in the CHO+PROT group would require that the glycogen content was higher by around 14 mmol·kg^-1^, which seems unlikely on isocaloric diets.

Blood glucose concentrations were reduced after the exhaustive exercise before the dietary interventions and at the end of the performance tests after the three dietary interventions. A decline in blood glucose normally stimulates glucagon secretion [44,45] and plasma glucagon was elevated at exhaustion prior to the diet interventions. During the first 2 h of the diet interventions, glucagon dropped in CHO and CHO+PROT in conjunction with the increase in plasma glucose concentration, as glucagon remained elevated in PLA where plasma glucose remained low. During the performance test (Day2), plasma glucagon increased in CHO+PROT and PLA as expected. Surprisingly plasma glucagon did not increase in CHO. Indeed, blood glucose decreased to a similar level at exhaustion among all dietary interventions and it is not obvious why glucagon remained low in CHO during the exhaustive exercise. FFA increased similarly during the performance test for all dietary interventions, but the increase in FFA was less in CHO compared to CHO+PROT and PLA after exercise, which may indicate that carbohydrate availability at exhaustion was higher in CHO compared to CHO+PROT and PLA.

Exhaustive exercise promotes protein degradation and protein degradation continues after exercise [12]. Intake of carbohydrate after exercise slows protein degradation [14], whereas a positive protein synthesis can be obtained when protein is ingested [17,19]. In the present study, subjects were in positive nitrogen balance after CHO+PROT despite higher plasma ornitine and citrulline during the first 2 h of recovery and higher total urea excretion. On the other hand, nitrogen balance was negative after CHO and PLA, and more negative in PLA than in CHO despite similar intakes of protein, supporting carbohydrate consumption immediately after exercise spares muscle protein by reducing protein degradation [14]. In the present study, the concentrations of BCAA and many other essential amino acids increased immediately after intake of CHO+PROT as expected [32], which prevents loss of amino acids from muscles [15]. Phenylalanine and tyrosine are not degraded in skeletal muscle and their release is used as a surrogate marker for protein degradation, but venous plasma data are difficult to interpret, and both amino acids increased in CHO+PROT whereas plasma phenylalanine remained similar in CHO and PLA.

Intake of protein immediately after exercise has consistently been shown to increase protein synthesis [16,17], and gene transcription is dramatically influenced by this protein intake [46]. It has been shown that protein intake in the early phase of recovery, as done in the present study, stimulates protein synthesis more pronounced than when provided later [19]. Amongst the BCAA, leucine plays a prominent role as it stimulates protein synthesis via activation of mTOR [47]. Importantly, intake of proteins together with carbohydrate after exercise increases activation of hypertrophic signalling molecules in skeletal muscle [20] and stimulates synthesis of both myofibrillar and mitochondrial proteins [18]. The positive nitrogen balance after CHO+PROT may have resulted from both reduced protein degradation and increased protein synthesis, whereas the negative nitrogen balance in CHO and PLA results from a higher protein degradation compared to protein synthesis. The negative nitrogen balance suggests a catabolic state, and the nitrogen balance status has previously been suggested to determine performance outcome after dietary interventions [29]. Our results, moreover, clearly indicate that a substantial amount of dietary protein is warranted soon after exhaustive exercise and for the remainder of the day. The daily protein requirement for endurance athletes has been reported to be 1.6 g · kg^-1^ [48], which is twice the recommended daily allowance for the average young adult. During the complete recovery period (18 h), CHO and PLA received 1.03 g protein · kg^-1^ and nitrogen balance was negative, whereas CHO+PROT received 1.83 g protein · kg^-1^ and nitrogen balance was positive, agreeing with previous findings [49].

We designed our study so that the workload (Watt_72%_) during exhaustive cycling was the same both days assuming that the physiological mechanisms causing fatigue during the initial bout of exhaustive exercise would determine performance capacity during the post exercise test. However, this assumption is not necessarily valid as the metabolism in recovering muscles may change. Even though subjects cycled to exhaustion at the same workload prior to and after the different dietary interventions, fatigue may have resulted from different mechanisms for the two performance tests. The mechanisms resulting in exhaustion during prolonged cycling at 72% of VO_2max_ is not well defined, but deletion of the mitochondrial branched-chain aminotransferase in mice skeletal muscle impairs endurance exercise capacity dramatically [50]. Branched chain amino acids are mainly metabolized in skeletal muscles [51] and protein intake immediately after exercise increases leucine oxidation [32]. Indeed, plasma valine was higher in CHO+PROT than in CHO prior to the performance test and it would have been attractive to speculate that mitochondrial function was better after intake of CHO+PROT compared to CHO. However, there were no significant differences in substrate selection during the performance test andthe concentrations of plasma lactate and amino acids did not differ between CHO and CHO+PROT. Skeletal muscles have a net uptake of glutamate during exercise [13] and glutamate is a major contributor to Krebs cycle intermediates, particularly α-ketoglutarate. Plasma glutamate decreased during the performance test (Day2), but the statistical power was low and there was no treatment effect. However, the fact that exhaustion occurred faster in PLA and CHO than in CHO+PROT indicates a faster decline in glutamate in PLA and CHO compared to CHO+PROT. Since glutamate is a major contributor to Krebs cycle intermediates we speculate that fatigue may have been associated with a decline in key Krebs cycle intermediates due to a decline in plasma glutamate.

In the present study, time to exhaustion was much longer prior to the dietary interventions despite the exercise intensities being similar for both cycling tests. The cycling was performed as 20 min intervals prior to the dietary intervention to reduce mental effort, but was continuous after dietary intervention, and therefore time to exhaustion cannot be compared directly. However, Hermansen et al. found similar glycogenolytic rates during continuous cycling and cycling at 20 min intervals [1], and we have in a pilot study seen that time to exhaustion is similar with intervals and continuous cycling (Jensen, unpublished). Our results, therefore, suggest that recovery of muscle function during the 18 h recovery period was incomplete even with high intake of protein.

Another interesting finding in the present study was that plasma uric acid increased after the exhaustive exercise prior to dietary interventions and more so in PLA compared to CHO and CHO+PROT during the recovery period. Uric acid is the product from AMP and GMP (pyridines) degradation and xanthine oxidase catalyses the final reaction where xanthine is converted to uric acid under formation of hydrogen peroxide (H_2_O_2_). Several studies have reported that uric acid increases after exhaustive exercise [52,53], but this is the first study to report that dietary intervention after exercise influences uric acid levels. Of note, plasma uric acid remained higher in PLA compared to CHO and CHO+PROT the day of the second cycling performance test and therefore nucleotide degradation may have been elevated during PLA. However, creatinine excretion was similar suggesting that degradation of phosphocreatine was unchanged and markers of muscle damage (CK, LDH and myoglobin) only varied minimally and were not influenced by the dietary interventions. These results suggest that recovery of performance was not linked to muscle damage or degradation of ATP/phosphocreatine system.

A major strength of the present study was that diet was controlled strictly during the whole recovery period and training and diet were controlled the last 24 h before the subjects entered the three dietary interventions. The present study was also randomized, double-blinded and conducted on well-trained subjects accustomed to forceful exercise and cycling training, which minimized the risk of lack of motivation and learning effects influencing the outcome. The study was demanding for the subjects with 6 exhaustive exercise sessions within 3 weeks, but the subjects remained highly motivated before all performance tests. Furthermore, motivation scores did not show a treatment or time effect and time to exhaustion was rather similar prior to the three interventions. A limitation of the study is that we did not take muscle biopsies and therefore there was no data on muscle glycogen or other metabolites/signalling molecules/mRNA, which could have helped explain differences in recovery performance. However, muscle biopsies were avoided to improve the quality and reproducibility of the performance tests. Another limitation in the present study was that a resting blood sample was not taken prior to the first exhaustive exercise bout or a fasted blood sample after a day without exercise.

In conclusion, intake of protein in combination with carbohydrate during the two first hours after exhaustive exercise results in a better and faster recovery of performance as compared to intake of carbohydrate only. The positive nitrogen balance observed in the CHO+PROT group provides evidence for an increased protein synthesis rate. Reducing carbohydrate intake during the recovery period, while maintaining protein intake, reduced time to exhaustion and caused a more negative nitrogen balance. Our results thus suggest that intake of protein immediately after exhaustive exercise stimulates recovery, which improves performance 18 h later. Ingestion of carbohydrate is also beneficial as opposed to fasting post exercise, potentially due to increasing muscle glycogen stores, reducing protein degradation or both.

## Supporting Information

S1 Fig Concentrations of organic acids in fasted condition the day after exhaustive exercise and diet interventions, and during and after the performance test. (TIFF)

S1 Table Intake of macro nutrients during the 18 h of recovery during the three dietary interventions. Subjects were supplied with standardized diet according to body weight.
(DOCX)

S2 Table Resting VO_2_, RER and carbohydrate oxidation during the first 2 hours recovery after ingestion of CHO, CHO+PROT and PLA. (DOCX)

S3 Table Plasma amino acids after exhaustion before diet intervention and fasting morning values after the three diet interventions. (DOCX)

S4 Table Plasma organic acids at exhaustion prior to the dietary interventions and the following morning (Day 2). (DOCX)

S5 Table Plasma LDH, CK and MYO after exhaustive exercise and during the recovery period and after the performance test. (DOCX)
