# Supplementary figures and images for: Intake of Protein Plus Carbohydrate during the First Two Hours after Exhaustive Cycling Improves Performance the following Day
Source: PLoS One. 2016 Apr 14;11(4):e0153229. doi: 10.1371/journal.pone.0153229 (PMC4831776; doi:10.1371/journal.pone.0153229)

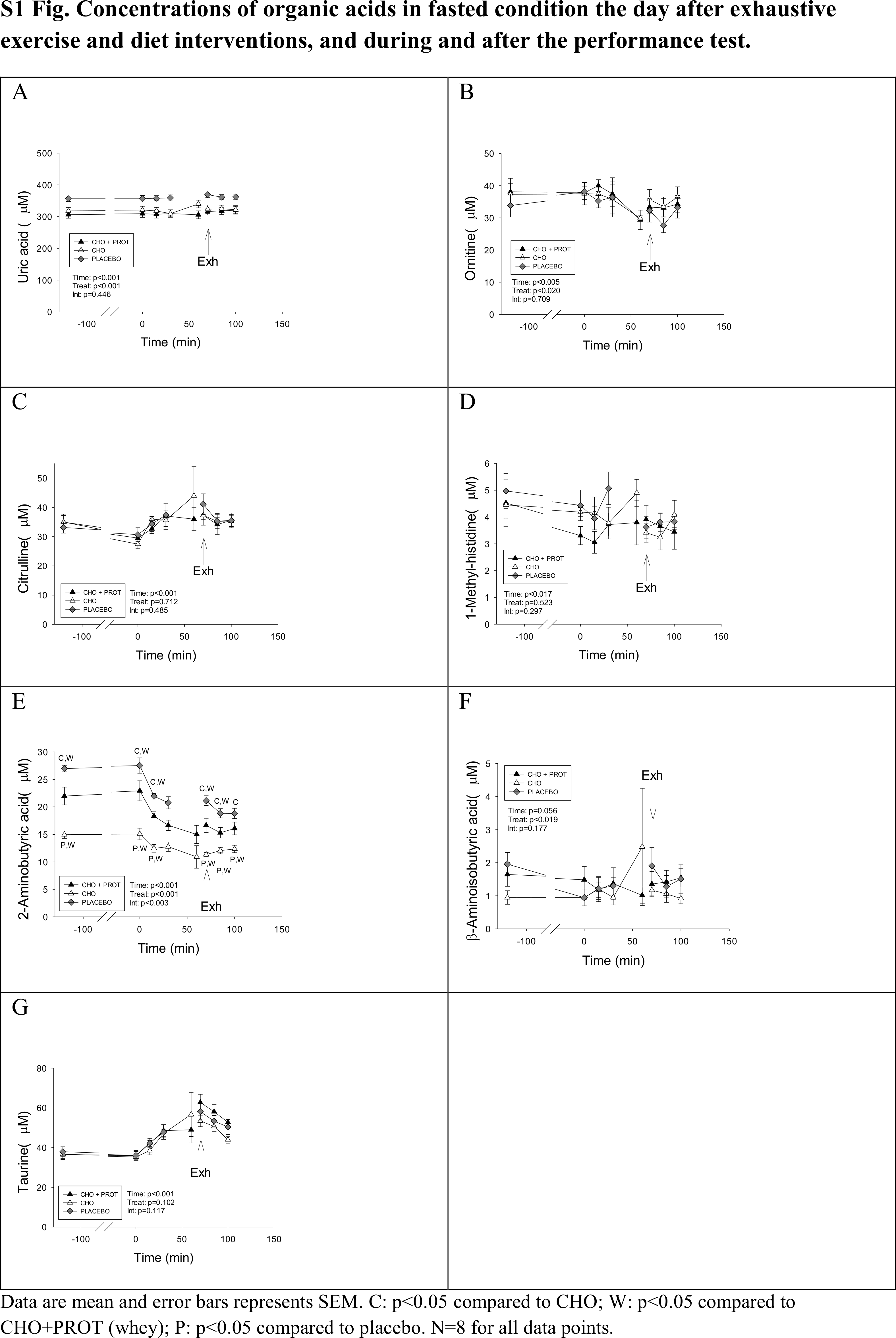

Supplement: S1 Fig — (TIFF) [file pone.0153229.s001.tiff]
