# Supplementary material for: Intake of Protein Plus Carbohydrate during the First Two Hours after Exhaustive Cycling Improves Performance the following Day
Source: PLoS One. 2016 Apr 14;11(4):e0153229. doi: 10.1371/journal.pone.0153229 (PMC4831776; doi:10.1371/journal.pone.0153229)
Supplement: S1 Table — Subjects were supplied with standardized diet according to body weight. (DOCX) [file pone.0153229.s002.docx]

**S1 Table. Intake of macro nutrients during the 18 h of recovery during the three dietary interventions.**

|  | **Carbohydrate** (g·kg^-1^) | **Protein** (g·kg^-1^) | **Fat** (g·kg^-1^) | **Energy** (kJ·kg^-1^) |
| --- | --- | --- | --- | --- |
| **CHO** | 7.55 | 1.03 | 0.72 | 175 |
| **CHO+PROT** | 6.75 | 1.83 | 0.72 | 175 |
| **PLA** | 3.95 | 1.03 | 0.72 | 113 |

Subjects were supplied with standardized diet according to body weight. See figure 1 for further information.
