# Supplementary material for: Intake of Protein Plus Carbohydrate during the First Two Hours after Exhaustive Cycling Improves Performance the following Day
Source: PLoS One. 2016 Apr 14;11(4):e0153229. doi: 10.1371/journal.pone.0153229 (PMC4831776; doi:10.1371/journal.pone.0153229)
Supplement: S2 Table — (DOCX) [file pone.0153229.s003.docx]

**S2 Table. Resting VO_2_, RER and carbohydrate oxidation during the first 2 hours recovery after ingestion of CHO, CHO+PROT and PLA.**

|  | **30 min** | **60 min** | **90 min** | **120 min** |
| --- | --- | --- | --- | --- |
| **VO_2_ (l·min^-1^)** |  |  |  |  |
| CHO | 0.36 + 0.02 | 0.34 + 0.02 | 0.33 + 0.01 | 0.33 + 0.01 |
| CHO+PROT | 0.38 + 0.01 ^P^ | 0.38 + 0.01 ^P,C^ | 0.37 + 0.01 ^P,C^ | 0.38 + 0.01 ^P,C^ |
| PLA | 0.33 + 0.02 | 0.33 + 0.02 | 0.32 + 0.02 | 0.33 + 0.01 |
| **RER (VCO_2_/VO_2_)** |  |  |  |  |
| CHO | 0.65 + 0.02 | 0.73 + 0.01 ^P^ | 0.77 + 0.01 ^P^ | 0.82 + 0.01 ^P^ |
| CHO+PROT | 0.64 + 0.02 | 0.71 + 0.01 ^P^ | 0.75 + 0.01 ^P^ | 0.79 + 0.01 ^P^ |
| PLA | 0.62 + 0.02 | 0.65 + 0.02 | 0.66 + 0.02 | 0.69 + 0.02 |
| **CHO oxidation (g·min^-1^)** |  |  |  |  |
| CHO | - | 0.03 + 0.01 ^P^ | 0.09 + 0.02 ^P^ | 0.17 + 0.02 ^P^ |
| CHO+PROT | - | 0.02 + 0.01 ^P^ | 0.07 + 0.01 ^P^ | 0.13 + 0.02 ^P^ |
| PLA | - | - | - | 0.02 + 0.01 ^#^ |

Values are means + SEM. n = 8. * Significantly different from values at 0 min (start of the time to exhaustion test) (p<0.05). † Significantly different from CHO (p<0.05). ^P^ Significantly different from PLA (p<0.05). ^C^ Significantly different from CHO (p<0.01). ^#^ After 120 min recovery, RER increased above 0.70 in some subjects in PLA; therefore the table shows some CHO oxidation at 120 min despite mean RER was below 0.70.
