# Supplementary material for: Intake of Protein Plus Carbohydrate during the First Two Hours after Exhaustive Cycling Improves Performance the following Day
Source: PLoS One. 2016 Apr 14;11(4):e0153229. doi: 10.1371/journal.pone.0153229 (PMC4831776; doi:10.1371/journal.pone.0153229)
Supplement: S3 Table — (DOCX) [file pone.0153229.s004.docx]

**S3 Table. Plasma amino acids after exhaustion before diet intervention and fasting morning values after the three diet interventions.**

| **Amino acid** | **Concentration (µM)** | | | |
| --- | --- | --- | --- | --- |
|  | **Exhaustion** | **CHO+PROT** | **CHO** | **PLA** |
| Alanine | 338.6±12.3 | 285.0±14.5 ^E^ | 275.8±17.8 ^E^ | 258.6±10.4 ^E^ |
| Arginine | 64.1±3.7 | 73.0±3.7 | 76.5±3.4 | 74.7±3.1 |
| Asparagine | 36.1±1.5 | 61.5±2.8 ^E^ | 57.4±3.3 ^E^ | 58.5±2.8 ^E^ |
| Glutamate | 24.9±2.7 | 25.1±1.6 | 27.6±2.6 | 25.8±3.1 |
| Glutamine | 493.5±12.7 | 710.0±22.4 ^E^ | 661.6±28.4 ^E^ | 675.6±25.0 ^E^ |
| Glycine | 144.2±3.7 | 201.0±6.2 ^E^ | 207.5±9.7 ^E^ | 197.5±6.6 ^E^ |
| Histidine | 67.1±3.9 | 88.1±6.0 ^E^ | 94.2±6.4 ^E^ | 91.0±7.0 ^E^ |
| Isoleucine | 76.1±3.1 | 92.0±3.9 ^E^ | 89.9±1.7 ^E^ | 96.7±2.5 ^E^ |
| Leucine | 132.2±3.8 | 165.6±6.6 ^E^ | 157.5±3.8 ^E^ | 172.5±6.4 ^E^ |
| Lysine | 116.3±4.3 | 176.8±7.2 ^E,C^ | 149.0±7.9 ^E^ | 159.5±7.2 ^E^ |
| Methionine | 13.1±0.4 | 17.0±1.1 | 16.0±1.8 | 15.8±0.9 |
| Phenylalanine | 48.3±1.4 | 55.1±1.2 ^E^ | 55.4±1.5 ^E^ | 56.9±2.6 ^E^ |
| Proline | 192.7±17.0 | 253.0±15.2 ^E^ | 219.4±11.1 | 213.7±12.4 |
| Serine | 71.9±1.4 | 109.1±3.9 ^E^ | 103.0±3.7 ^E^ | 109.5±5.2 ^E^ |
| Threonine | 94.1±2.8 | 150.1±6.4 ^E,C,P^ | 121.8±2.9 ^E,W^ | 124.4±6.2 ^E,W^ |
| Tryptophan | 40.6±2.0 | 49.2±1.0 ^E^ | 50.2±1.4 ^E^ | 46.8±1.3 ^E^ |
| Tyrosine | 67.2±1.7 | 65.6±2.0 | 67.9±1.5 | 62.6±1.8 |
| Valine | 241.0±8.8 | 261.3±9.4 ^E,C^ | 234.9±4.6 ^W,P^ | 268.6±7.5 ^E,C^ |

Data are mean ± SEM. Concentrations at exhaustion are means of the three exhaustion tests before the diet intervention (no differences in any of the amino acids between the three tests). Data are compared with ANOVA with LSD as post hoc test. N=8 for all data set. E: p<0.05 compared with Exhaustion; C: p>0.05 compared CHO; W: p<0.05 compared to CHO+PROT; P: p<0.05 compared to PLA.
