# Supplementary material for: Intake of Protein Plus Carbohydrate during the First Two Hours after Exhaustive Cycling Improves Performance the following Day
Source: PLoS One. 2016 Apr 14;11(4):e0153229. doi: 10.1371/journal.pone.0153229 (PMC4831776; doi:10.1371/journal.pone.0153229)
Supplement: S4 Table — (DOCX) [file pone.0153229.s005.docx]

**Table S4. Plasma organic acids at exhaustion prior to the dietary interventions and the following morning (Day 2).**

| **Plasma organic acids** | **Concentration (µM)** | | | |
| --- | --- | --- | --- | --- |
|  | **Exhaustion** | **CHO+PROT** | **CHO** | **PLA** |
| Ornithine | 30.6±2.5 | 38.1±4.2 ^E^ | 37.3±3.4 ^E^ | 36.4±3.3 ^E^ |
| Citrulline | 35.7±2.1 | 35.0±2.7 | 35.0±2.2 | 33.1±1.9 |
| 1-Methylhistidine | 6.6±1.3 | 6.6±1.3 | 4.5±0.5 | 5.0±0.7 |
| Taurine | 76.7±6.1 | 36.5±2.0 ^E^ | 36.7±2.7 ^E^ | 37.9±2.5 ^E^ |
| 2-Aminobutyric acid | 10.0±0.7 | 22.0±1.6 ^E,C,P^ | 15.0±0.7 ^E,W,P^ | 27.0±0.6 ^E,W,P^ |

Data are mean ± SEM. Concentrations at exhaustion are means of the three exhaustion tests before the diet intervention (no differences in any of the amino acids between the three tests). Data are compared with ANOVA with LSD as post hoc test. N=8 for all data set. E: p<0.05 compared with Exhaustion; C: p>0.05 compared CHO; W: p<0.05 compared to CHO+PROT; P: p<0.05 compared to PLA.
