# Supplementary material for: Intake of Protein Plus Carbohydrate during the First Two Hours after Exhaustive Cycling Improves Performance the following Day
Source: PLoS One. 2016 Apr 14;11(4):e0153229. doi: 10.1371/journal.pone.0153229 (PMC4831776; doi:10.1371/journal.pone.0153229)
Supplement: S5 Table — (DOCX) [file pone.0153229.s006.docx]

**S5 Table. Plasma LDH, CK and MYO after exhaustive exercise and during the recovery period and after the performance test.**

|  | **Exhaustion** | **120 min** | **Morning (Day 2)** | **Start TTU** | **Exhaustion TTU** |
| --- | --- | --- | --- | --- | --- |
| **LDH (U·L^-1^) *** |  |  |  |  |  |
| CHO | 176.4+12.23 | 157.8+12.6 | 144.7+11.9 | 144.7+11.4 | 162.7+10.9 |
| CHO+PROT | 163.4+11.1 | 148.4+10.7 | 134.5+8.1 | 137.1+9.4 | 155.4+11.6 |
| PLA | 180.1+7.1 | 158.3+9.1 | 135.7+9.1 | 143.9+10.5 | 163.7+15.2 |
| **CK (U·L^-1^)** |  |  |  |  |  |
| CHO | 361.6+140.8 | 324.4+124.0 | 278.6+100.5 | 269.4+99.3 | 289.6+101.4 |
| CHO+PROT | 193.0+41.7 | 171.3+34.7 | 157.3+33.5 | 150.8+31.5 | 175.4+37.3 |
| PLA | 296.0+93.7 | 264.6+86.8 | 286.8+117.3 | 286.8+116.0 | 320.8+130.9 |
| **MYO (µg·L^-1^) *** |  |  |  |  |  |
| CHO | 66.2+16.0 | 81.3+18.0 | 57.2+13.8 | 55.4+17.4 | 64.5+19.9 |
| CHO+PROT | 48.4+7.82 | 63.3+9.7 | 44.3+4.9 | 39.3+4.3 | 50.8+7.9 |
| PLA | 66.1+13.4 | 75.9+13.7 | 62.5+13.7 | 58.1+12.8 | 67.0 + 15.5 |

Data are mean ± SEM. N=8 for all data points. LDH: Lactate dehydrogenase; CK: Creatine kinase; MYO: Myoglobin. *: Repeated measurement showed time effect (p<0.01) for LDH and MYO, but no treatment effect or interaction.
